# The strategies and advances of mRNA translation booster

**DOI:** 10.1016/j.ajps.2025.101090

**Published:** 2025-09-02

**Authors:** Yingying Shi, Kedong Sun, Yilong Hu, Zeliang Lou, Yi Wang, Jian You

**Affiliations:** a College of Pharmaceutical Sciences, Zhejiang University, Hangzhou 310058, China; b State Key Laboratory for Diagnosis and Treatment of Infectious Diseases, Hangzhou 310006, China; c The First Affiliated Hospital, College of Medicine, Zhejiang University, Hangzhou 310000, China; d Jinhua Institute of Zhejiang University, Jinhua 321299, China

**Keywords:** mRNA expression, Immunogenicity, Translation boosters, Nucleic acid therapeutics

## Abstract

The therapeutic efficacy and safety of mRNA-based drugs in immunological and nonimmunological applications are critically dependent on the translated protein yield, which requires precise modulation of mRNA expression kinetics. Among the factors influencing mRNA translation, immunogenicity and stability are pivotal in determining the longevity of protein production. Current optimization strategies have integrated (1) molecular engineering (*e.g.*, modified nucleotides), (2) advanced delivery systems (*e.g.*, lipid nanoparticles), and (3) adjuvant drug synergy. This review focuses on co-delivered adjuvant drugs and introduces the concept of "mRNA translation boosters" for the first time. mRNA translation boosters are classified as small-molecule compounds and macromolecular agents that improve translational fidelity through mechanisms including blockade of pattern recognition receptors, modulation of inflammatory cascades, facilitation of endosomal escape, and protection against enzymatic degradation. As clinically validated with COVID-19 mRNA vaccines, these boosters have now demonstrated expanded utility in gene editing therapies and protein replacement applications. This review addresses the immunological challenges encountered during mRNA transfection and translation while summarizing existing mRNA translation boosters that optimize protein expression kinetics. By establishing a mechanistic framework for booster selection and employment, this work provides translational guidance for advancing nucleic acid therapeutics towards their maximum clinical potential.

## Graphical abstract

Here, we provide a systematic review of the knowledge pertaining to the immune response pathways triggered by *in vitro*-transcribed mRNA transfection, summarize strategies for optimizing mRNA expression and their corresponding mRNA translation boosters, and discuss the challenges that need to be addressed in this field moving forwards.

**Figure:**
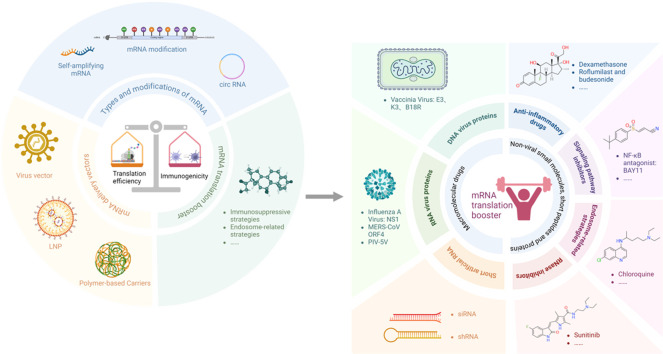

## 1 Introduction

In 2021, the U.S. Food and Drug Administration (FDA) approved the first mRNA-based vaccine formulations developed by Pfizer/BioNTech and Moderna [[1], [2], [3]] to combat the global spread of COVID-19, reigniting interest in mRNA therapies [4]. However, the origin of this disruptive technology can be traced back to a foundational discovery made thirty years ago. In 1990, the groundbreaking research of Wolff and colleagues was the first to demonstrate that naked synthetic mRNA administered via intramuscular injection could be successfully expressed *in vivo* [5,6]. This proof-of-principle achievement has opened up a whole new dimension for the field of gene therapy [7]. However, researchers soon realized that there were still two insurmountable technical barriers on the path from initial laboratory discovery to practical clinical application. On the one hand, the inherent instability of mRNA molecules makes it difficult for them to maintain their pharmacological activity over an extended period [8]. On the other hand, the body's innate immune response may lead to a series of safety risks upon administration [9,10]. In recent years, with the continuous maturation of rational design strategies such as mRNA nucleotide modification [11] and codon optimization, as well as the successful development of novel delivery systems such as lipid nanoparticles (LNPs) [[12], [13], [14]], these bottlenecks, which have long hindered technological transformation, have been gradually overcome. This profound transformation of the technological paradigm has brought renewed interest into mRNA therapies, accelerating their transition from laboratory research to clinical application. Recently, mRNA therapies have shown extremely broad application prospects in multiple medical fields, including infectious disease prevention and control [15,16], tumor immunotherapy [17,18], etc.

The mRNA or its carrier is internalized by cells through endocytosis, via the formation of endosomal vesicles. Within these endosomal vesicles, mRNAs may be recognized by Toll-like receptors (TLRs), leading to their degradation. Therefore, the process by which mRNAs escape from endosomal vesicles into the cytoplasm, known as endosomal escape, is crucial for their functional expression. Once an mRNA successfully escapes and enters the cytoplasm, ribosomes recognize and bind to the mRNA, subsequently synthesizing specific proteins on the basis of the genetic information it carries. This translation process constitutes the primary function of mRNAs and is central to their therapeutic potential [19]. Many synthesized proteins, after translation, undergo further posttranslational modifications in the endoplasmic reticulum, including protein folding and glycosylation, to ensure that they acquire full biological activity and stability. The developed mRNA technology platforms are rather diverse. Currently, the main types of mRNA technology used include classical nonreplicating mRNA (NRM), self-amplifying mRNA (sa-RNAs), trans-amplifying mRNA (ta-RNA) and circular RNA (circ-RNA). Each type of mRNA platform has its own unique advantages and limitations, as described below.

NRM is the most extensively studied RNA platform. An mRNA molecule is composed of five essential elements: 5′ cap, 5′-untranslated region (5′-UTR), open reading frame (ORF), 3′-UTR and 3′ poly(A) tail. Each of these elements plays a crucial role in the mRNA function as a template for protein synthesis [20]. Specifically, the 5′ cap is a methylated guanosine structure that facilitates pre-mRNA splicing, mRNA export from the nucleus to the cytoplasm, the initiation of translation, and protection against degradation by exonucleases [21,22]. The 5′-UTR serves as a platform for the assembly of the transcription machinery and can enhance or hinder translation [[23], [24], [25]]. The ORF encodes the sequence that determines the functional characteristics of the vaccine [26,27]. The 3′-UTR influences mRNA localization, stability, and translation efficiency [[28], [29], [30]]. The poly(A) tail, located at the 3′ end of the mRNA, is recognized by poly(A) binding proteins (PABPs) to form a translation complex and protects the mRNA from exonuclease degradation, thereby increasing its stability and prolonging its lifespan. These factors contribute to increased protein yield [31,32]. However, in general, the *in vivo* half-life of NRMs is relatively short, and the amount of antigen expressed is relatively low.

Sa-RNAs are extension of NRMs [33,34]. These platforms share the same basic structure, but sa-RNAs include additional nonstructural proteins (NSPs) derived from alphaviruses (NSP1, NSP2, NSP3 and NSP4) and the alphavirus subgenomic promoter (SGP) region [35]. This allows sa-RNAs to self-replicate via replicases located downstream of the 5′ UTR. Once sa-RNAs enter the cytoplasm of target cells, they initiate protein translation, which primarily involves the NSPs located upstream of the main protein-coding region. NSPs form an early replication complex, producing complementary RNA strands using the sa-RNAs template. Subsequently, NSP polyproteins are cleaved into individual proteins, forming late replication complexes. These late replication complexes synthesize copies of sa-RNAs on the RNA template, thus increasing the initial quantity of *in vitro*-transcribed mRNA (IVT mRNA) copies. The SGP facilitates the initiation of transcription of the gene of interest (GOI) by bypassing the sequences encoding viral NSP proteins [36]. This mechanism promotes the formation of mRNA copies containing only the GOI, which are then translated. Compared with NRM drugs, the replication mechanism of sa-RNAs reduces the required dose by 30- to 1,000-fold [37]. Consequently, the lower dosage requirements of sa-RNAs lead to reduced immunogenicity, increased safety, and a prolonged duration of action, thereby contributing to the generation of a robust immune response and necessitating less raw materials for synthesis [38,39]. However, viral NSPs can impact host cells and trigger excessive immune activation, and the intracellular double-stranded RNA (dsRNA) produced during replication can activate innate immunity and interfere with protein translation [40,41].

To address several of the issues associated with the sa-RNAs platform, researchers developed a ta-RNA platform based on a dual-mRNA system [42]. Ta-RNA is a special form of sa-RNAs, in which the viral sequences, NSPs, and GOI are present in independent mRNA molecules but function synergistically [42,43]. This design retains the advantages of sa-RNAs while circumventing some of their drawbacks. For example, in sa-RNAs, since the viral sequences, NSPs, and GOI are typically located on the same mRNA molecule, the length of the GOI is subject to certain limitations. An overly long GOI may affect the stability, translation efficiency, and replication of the mRNA. In contrast, ta-RNAs separate these components, overcoming the length limitation of the GOI. Thus, ta-RNAs can carry larger and more complex target genes, making it possible to express large or multi-subunit therapeutic proteins [44]. However, ta-RNAs require at least two different RNA molecules (one encoding the replicase and the other encoding the GOI), which results in increased immunogenicity [45]. Currently, research on ta-RNA technology is still in its early stages. Nevertheless, there have been several remarkable cutting-edge advancements in fields such as vaccines [46] and gene therapy [44].

In 2015, the discovery of translatable circ-RNA in *Drosophila melanogaster* overcame the cognitive limitations of traditional concepts regarding the structure and function of RNA. This technique is similar to a pebble thrown into the scientific research field, stirring ripples layer by layer and revealing its enormous potential as a vaccine platform[47]. Circ-RNAs have a unique circular structure that protects them from degradation by exonucleases. Exonucleases usually start removing nucleotides from the ends of RNA molecules, while circ-RNAs have no free 5′-end or 3′-end; thus, it is similar to putting on a bulletproof vest for the RNA molecule, effectively resisting the attack of exonucleases. This stability enables circ-RNAs to survive longer in cells and have a longer half-life [48,49]. In contrast, linear mRNA can be easily recognized and rapidly degraded by exonucleases, and their stability *in vivo* is relatively poor. However, the circ-RNAs preparation process is relatively complex with a high degree of technical difficulty. Currently, circ-RNAs are synthesized mainly through *in vitro* transcription and cyclization reactions. However, these methods result in low yield, and ensuring the quality and uniformity of the synthesized circ-RNA is difficult. During cyclization, issues such as residual linear RNA and incomplete cyclization may occur, affecting the stability and function of the product circ-RNAs. In addition, the large-scale production of circ-RNAs faces challenges such as high costs and difficulties in process optimization, which, to a certain extent, limit the widespread application of circ-RNAs. It is believed that in the future, the difficulties in circ-RNA production technology can be overcome, enabling circ-RNAs to play a greater role in the biomedical field [[50], [51], [52]].

**Fig. 1:**
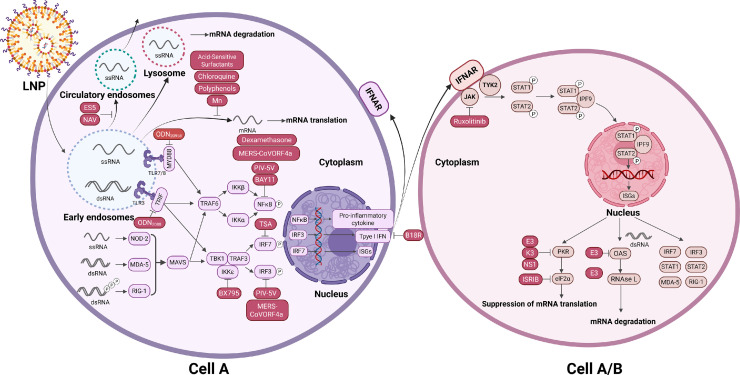
Innate immune response to the intracellular delivery of IVT mRNA and associated inhibitory strategies. Synthetic mRNAs, which may contain the byproduct dsRNA, are recognized by a variety of PRRs. These include TLR3 and TLR7/8 within endosomes, as well as RIG-I, melanoma differentiation-associated protein 5 (MDA-5) and nucleotide binding oligomerization domain containing 2 (NOD-2) within the cytoplasm. Each PRR interacts with specific adapter molecules that recruit signaling molecules, thereby activating downstream TFs, including IRF3, IRF7 and NF-κB. IRF3 and IRF7 regulate the expression of IFN, whereas NF-κB is responsible for controlling the production of proinflammatory cytokines. Type I IFNs bind to IFNARs through autocrine or paracrine mechanisms, leading to the activation of JAK1 and TYK2, which in turn phosphorylate STAT1 and STAT2. Phosphorylated STAT1 and STAT2 combine with IRF9 to form the transcriptional activation complex ISGF3. ISGF3 activates hundreds of ISGs, including genes encoding antiviral effector proteins such as OAS and PKR. This ultimately leads to inhibited RNA translation, increased of RNA degradation, and further amplification of the aforementioned immune response processes. This graphic was created with BioRender.com.

At present, the aforementioned mRNA platforms have been widely applied in both preclinical and clinical research, encompassing immunotherapy fields such as vaccination [53,54], as well as non-immunotherapy fields, such as protein replacement therapy [55,56], gene editing [[57], [58], [59], [60]], and stem cell reprogramming [[61], [62], [63], [64]]. However, the development and application of mRNA therapies still face numerous challenges, particularly regarding their low transfection efficiency [65] and poor safety [10,66]. This is due to the immunogenicity of IVT mRNA, that is, the IVT mRNA triggers an innate immune response when it enters mammalian cells [67,68] (Fig. 1). Although the intrinsic immunostimulation of mRNAs offers advantages in immunotherapy applications by enhancing vaccine efficacy [61,[69], [70], [71], [72]], this process is highly complex, and requires a delicate balance among various factors [9]. Overactivation of innate immunity can lead to flu-like symptoms and even autoimmune diseases, posing significant risks [[73], [74], [75]]. In non-immunotherapy applications, the immune response induced by mRNAs can cause the transfected cells and neighboring cells to become hypersensitive to subsequent mRNA exposure, leading to cell damage and death [76]. This immune defense mechanism significantly reduces delivery efficiency, inhibits protein translation, and is a major factor leading to the decreased intracellular stability of mRNA therapies, reduced translational robustness, limited continuity, and increased safety risks [77]. Therefore, regulating mRNA-mediated immune activation is crucial for controlling their ultimate immunogenicity and translational efficacy. Understanding the intrinsic mechanisms of mRNA immune activation is fundamental to achieving its regulation. In mammalian cells, synthetic mRNAs, exogenous single-stranded RNAs (ssRNAs), and immunostimulatory byproducts or contaminants produced during IVT mRNA synthesis [78] are recognized by pattern recognition receptors (PRRs) as the danger signals known as pathogen-associated molecular patterns (PAMPs) [79,80].

In addition, controlling activation of the innate immune system is crucial for preventing excessive immune responses, such as cytokine storms. The innate immune system rapidly recognizes PAMPs or damage-associated molecular patterns (DAMPs) through PRRs, such as TLRs and NOD-like receptors (NLRs), and activates pathways such as the nuclear factor kappa-light-chain-enhancer of activated B cells (NF-κB) pathway and the interferon (IFN) pathway. This process leads to the release of proinflammatory cytokines, such as interleukin-6 (IL-6), TNF-α and IL-1β, and chemokines, which recruit immune cells to eliminate pathogens. When infectious (such as severe COVID-19 and influenza) or noninfectious factors (such as CAR-T therapy and trauma) cause dyshomeostasis of the immune system, the cascade amplification effect of large amounts of cytokines, such as IL-6, IFN-γ, and granulocyte‒macrophage colony‒stimulating factor (GM‒CSF), triggers cytokine storm syndrome (CSS), leading to vascular leakage, ischemic organ damage, and multiple organ failure. Controlling the activation of the innate immune system is the core step in preventing cytokine storms, which requires a balance between suppressing excessive inflammation and maintaining immune defense.

PRRs play pivotal roles in initiating the innate immune response. The key PRR families include the TLRs, NLRs and RIG-I-like receptors (RLRs). These receptor classes exhibit distinct PAMP recognition specificities and downstream signaling mechanisms, resulting in unique modes of innate immune activation. TLRs localize to both the plasma and endosomal membranes of immune cells. Plasma membrane TLRs such as TLR4 recognize lipids and lipoproteins. Upon PAMP engagement, they recruit downstream signaling molecules via myeloid differentiation factor 88 (MyD88), activate NF-κB and upregulate proinflammatory cytokines to induce inflammatory responses. Endosomal TLR3 [[81], [82], [83], [84]] and TLR9 [[85], [86], [87]] specifically recognize dsRNA and dsDNA, respectively. For example, TLR3 triggers IFN-β production through TIR-domain-containing adapter-inducing IFN-β (TRIF), activating IFN regulatory factors 3 and 7 (IRF-3/7) to promote type I IFN expression, which is critical for antiviral immunity [88,89].

RLRs are ubiquitously expressed in the cytoplasm of immune and nonimmune cells. Cytoplasmic retinoic-acid-inducible gene I (RIG-I) detects short dsRNAs and ssRNAs with 5′-triphosphate groups. Ligand binding induces conformational changes in the caspase activation and recruitment domain (CARD) of RIG-I, enabling its interaction with the mitochondrial antiviral signaling protein (MAVS) [[90], [91], [92], [93]]. This complex activates the TANK binding kinase 1 (TBK1), which phosphorylates IRF-3 to drive type I IFN transcription. In contrast to TLR pathways, RIG-I signaling rapidly detects cytoplasmic viral RNA, enabling the early activation of immune defence [94,95]. NLRs function within the cytoplasmic compartment, detecting microbial components such as peptidoglycan, flagellin, toxins, and ATP. NLRP3 serves as a prototypical sensor, forming inflammasome complexes with apoptosis-associated speck-like protein containing a caspase recruitment domain (ASC) and caspase-1 in response to cellular stress signals (*e.g.*, potassium ion efflux and reactive oxygen species accumulation). Activated caspase-1 processes pro-IL-1β into its mature form, mediating inflammatory responses. Unlike the TLR/RLR pathways, which are focused on IFN induction, NLR signaling uniquely regulates inflammasome activation to control inflammation initiation and resolution [[96], [97], [98]].

Collectively, PRR engagement with PAMPs (including immunostimulatory byproducts such as dsRNA/DNA transcripts) initiates signaling cascades through adaptor molecules, activating the transcription factors NF-κB, IRF-3 and IRF-7. These factors translocate to the nucleus to upregulate type I IFN and proinflammatory cytokine gene expression [80,99]. Notably, IFNs not only enhance innate and adaptive immune responses but also play crucial roles in mediating mRNA degradation, the termination of translation, and the induction of apoptosis [100,101]. The produced type I IFNs are transported to the extracellular space, where they bind to IFN-α/β receptors (IFNARs) on the surface of stimulated and neighboring cells via autocrine or paracrine signaling. This interaction activates the Janus kinase (JAK)-signal transducer and activator of transcription (STAT) pathway, forming a transcriptional activator complex called IFN-stimulated gene factor 3 (ISGF-3), which is composed of phosphorylated STAT1, phosphorylated STAT2, and IRF-9 [[102], [103], [104]]. The ISGF-3 complex translocates to the nucleus and binds to the IFN-stimulated response element (ISRE) in the promoter region, initiating the transcription of IFN-stimulated genes (ISGs) [105]. Some ISGs encode proteins directly involved in innate immune pathways, such as PRRs and transcription factors (TFs), which serve as positive feedback regulators of IFN production [106]. This makes cells more sensitive to subsequent nucleic acid exposure, leading to potential cell damage and even death. Additionally, several ISGs encode cytoplasmic IFN-induced, dsRNA-dependent effector proteins with potent antiviral activity, including protein kinase dsRNA-dependent (PKR), 2′−5′-oligoadenylate synthetase (OAS), and adenosine deaminase acting on RNA (ADAR) [107]. Once it detects dsRNA, PKR phosphorylates the α subunit of eukaryotic initiation factor 2α (eIF2α), halting general mRNA translation and inhibiting protein synthesis [[108], [109], [110], [111]]. Concurrently, dsRNA activates OASs, which use ATP to generate 2′−5′-oligoadenylate (2–5A) [112]. Then, 2–5A oligomers activate the endoribonuclease RNase L, leading to mRNA cleavage and degradation [[113], [114], [115]]. On the other hand, exposure to dsRNA induces the catalytic activity of ADAR enzymes, which convert adenosine to inosine through deamination at specific sites. This results in mismatched base pairing, mRNA instability, and alterations in the amino acid sequences of the encoded proteins [116,117].

Given the significant detrimental effects of IFN induction on protein production, extensive efforts have been made to design mRNA molecules that avoid TLR detection and prevent IFN induction. These efforts include structural and chemical modifications of mRNA [11,118], the use of appropriate delivery systems [[119], [120], [121]], and mRNA coadministration with certain regulatory molecules [122,123]. On the one hand, innovations at the mRNA level, such as structural and sequence optimizations (cap modifications, poly(A) tails, noncoding regions, and codon replacements) [[124], [125], [126], [127]], and the incorporation of modified nucleotides [[128], [129], [130], [131], [132]] (such as pseudouridine [Ψ], 1-methylpseudouridine [m1Ψ], 5-methylcytidine [m5C], 6-methyladenosine [m6A], and 5-methyluridine [m5U]) [128], have greatly reduced mRNA immunogenicity. On the other hand, in terms of delivery systems, extensive research has been conducted on both viral [133] and nonviral vectors [14,134,135] to improve their efficiency and safety. Furthermore, efforts have also been made in production and purification processes to remove immunostimulatory impurities (especially dsRNA, Ψ, m1Ψ, m5C, m6A and m5U) [78,[136], [137], [138], [139], [140]].

However, both of the aforementioned strategies often require substantial design work from scratch, which involves significant resource investment. The combined use of regulatory drugs and mRNAs can alleviate this problem. For example, a series of drugs, including small molecules and macromolecular proteins/peptides, have been identified for this purpose. These drugs can increase the expression efficiency of therapeutic mRNAs through strategies such as promoting endosomal escape, blocking PRRs (*i.e.*, the initiation of immunogenicity), interfering with downstream signaling cascades (*i.e.*, the immunogenicity process), and directly inhibiting inflammatory effector molecules (*i.e.*, the consequences of immunogenicity). In this review, drugs with the above capacity are referred to as mRNA translation boosters. Owing to the lack of standardized experimental protocols and measurement standards in these studies, some experimental results may be contradictory. Therefore, we will focus on introducing experimental protocols from the perspectives of immunosuppression and translation enhancement, providing a reference for future research in this field.

## 2 Macromolecular drugs

### 2.1 Natural viral proteins

**Fig. 2:**
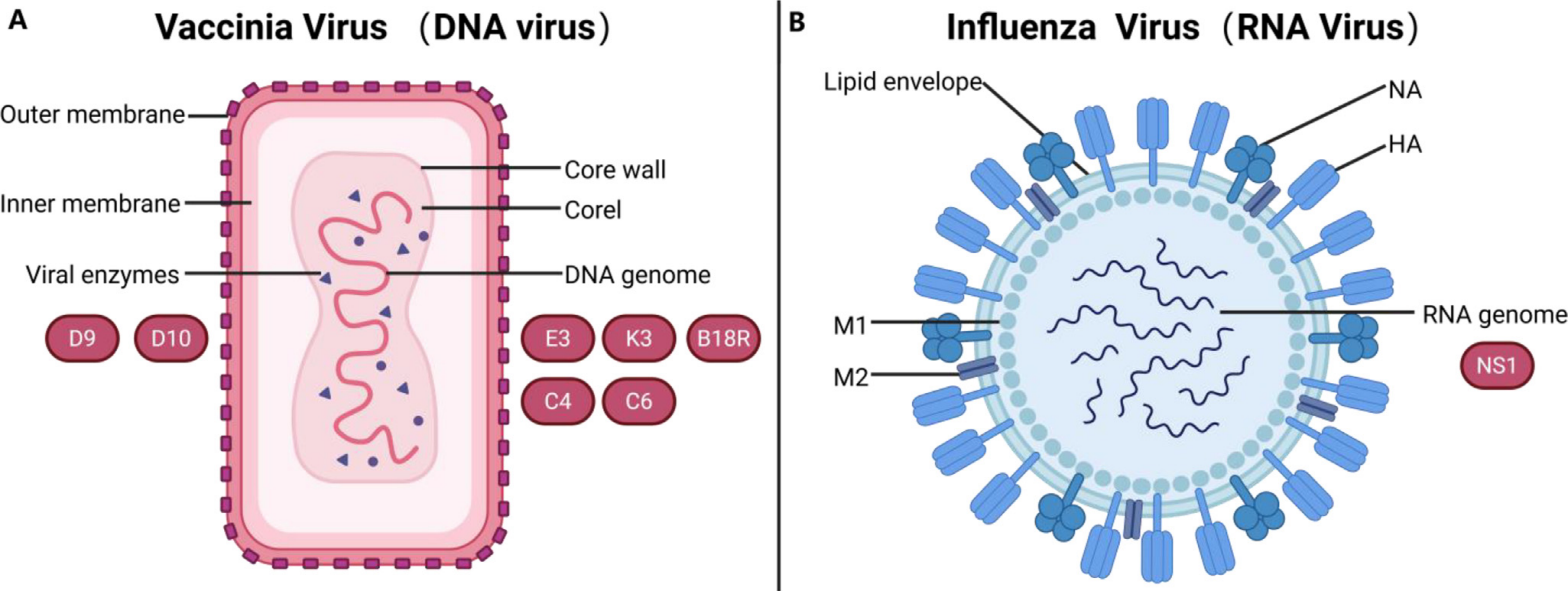
Structural analysis of DNA and RNA viruses and inhibition of immune response-related proteins. (A) Classic structure of DNA viruses, exemplified by vaccinia virus. The virus envelope is composed of a lipid bilayer, with an outer surface decorated by randomly arranged tubular components. These tubular components encase the core, which contains the DNA genome and viral enzymes such as D9 and D10. The genome can be edited to produce a variety of proteins, including E3, K3, B18R, C4 and C6. These proteins aid the virus in evading the host's innate immune response, thereby enhancing the virus's replication and transmission capabilities. (B) Classic structure of RNA viruses, exemplified by influenza virus. The virion is enveloped by two abundant membrane proteins, HA and NA, as well as a small amount of M2 ion channel protein. The M1 protein is located beneath the lipid membrane, providing structural support for the virion. The genome consists of multiple segments of single-stranded negative-sense RNA, encoding a variety of proteins, including structural proteins required for viral replication and NSPs such as NS1 that regulate viral replication, transcription, and interfere with the host's innate immune response. The graphic is created with BioRender.com.

Both DNA and RNA viruses consist of a core of genetic material and an outer shell. Over the course of evolution, viruses have developed diverse strategies to evade immune detection and downregulate induced responses [[141], [142], [143], [144], [145], [146]]. Thus, harnessing these immune-evasive viral proteins to simulate the virus's immune-evasion capabilities for the application in mRNA translation presents an attractive strategy (Fig. 2).

#### 2.1.1 Vaccinia Virus: EKB

##### 2.1.1.1 Overview of the Vaccinia Virus

The poxviridae are the most structurally complex class of DNA viruses known to date. Poxvirus particles exhibit an elliptical or brick-shaped structure, with lengths ranging approximately from 200 to 400 nm and an aspect ratio of 1.2–1.7. The membrane consists of a typical 50–55 nm lipid bilayer enveloping the core, with the outer surface adorned with randomly arranged tubular components. However, for certain virus strains and specific cell infections, virus particles can acquire an additional lipid bilayer with a unique chemical composition, termed the envelope [147]. The phospholipids contained in this envelope are approximately twice that of unenveloped virus particles. Extensive research indicates that antigens on the virus envelope can elicit an immune response, thus protecting the host against poxvirus attacks [148,149].

Cowpox virus (CPXV), belonging to the Poxviridae family, derives its name from its association with pustular lesions on the teats of cows and the hands of milkmaids. Vaccinia virus (VACV), based on CPXV, is a widely used laboratory model for poxviruses, employed in the production of smallpox vaccines, serving as a valuable expression vector, immunological tool, and a well-established model system for studying virus-host interactions [149]. Most of the studies summarized in this paper use VACV as the model.

VACV replicates in the cytoplasm and possesses a large dsDNA genome and complex virus particles. Approximately half of the VACV genes are non-essential for virus replication in cell culture but encode a series of immunomodulatory proteins, which almost antagonize the host IFN response at every stage of infection. Specifically, the *D9* and *D10 helicase proteins* are part of its strategy to inhibit cellular translation and promote viral protein synthesis [[150], [151], [152]]; the *dsRNA-binding protein E3* can prevent RNA polymerase III from sensing viral DNA, thus inhibiting IFN production, and can also inhibit activation of the PKR and OAS/RNase L pathways [[153], [154], [155]]; the *K3 protein*, structurally similar to eIF2α, competes with eIF2α for PKR phosphorylation, thereby preventing inhibition of protein synthesis [156,157]; *C4* and other *Cowpox-derived proteins* block NF-κB activation at almost every step of the NF-κB pathway [158,159]; the *C6 protein* prevents TBK1-driven activation of IRF-3 and IRF-7 [[160], [161], [162]]; and the *B18* and *B8 proteins* act as decoy receptors, neutralizing type I and type II IFN [163,164]. In theory, all the aforementioned proteins have the potential to enhance mRNA translation efficiency. In current practical applications, the most commonly used proteins are *B18R, E3* and *K3*.

##### 2.1.1.2 B18R in the field of mRNA translation

B18R is a 60–65 kDa glycoprotein, functioning as an IFN decoy receptor that neutralizes type I IFNs [165,166]. It has been demonstrated to inhibit the activation of IFNAR and subsequent pathway signaling during mRNA-based reprogramming, enhancing cell viability [167]. For instance, Beisser et al. [168] validated the efficacy of B18R in the transfection process of green fluorescent protein (GFP) sa-RNAs into human foreskin fibroblasts (HFF) using RNAiMAX, an efficient transfection reagent. Specifically, the reprogramming mRNA mixture consisted of 0.8 µg of octamer-binding transcription factor (OCT4) mRNA, sex-determining region of Y chromosome (SRY)-box transcription factor 2 (SOX2) mRNA, and other TF mRNAs (in ratios of 1:1:1:1:1:1 or 3:1:1:1:1:1:1), supplemented with 0.2 µg B18R and 0.4 µg miRNAs 302a-d and 367 (each at 0.4 µM). Experimental results showed that B18R could inhibit >90% of OAS1 induction, indicating its sufficient release from transfected cells to neutralize secreted IFN.

From a genetic perspective, Drews et al. [169] did a similar study. They evaluated B18R's ability to prevent upregulation of various innate immune response-related genes during mRNA complex delivery using real-time quantitative polymerase chain reaction (qRT-PCR). Specifically, HFF cells were first transfected with a complex of 4 µg GFP mRNA and 4 µl Lipofectamine RNAiMAX (LF), followed by transfection with an equal amount of mRNA mixture encoding TFs (such as OCT4, SOX2, Kruppel-like factor 4 (KLF4) and cellular myelocytomatosis oncogene (c-MYC)). The results indicated that although B18R exhibited inhibitory effects on the innate immune response activated after non-viral mRNA transfection, it did not induce downregulation of immune response-related genes. However, the fact that B18R can inhibit the innate immune response activated after mRNA transfection does not mean that it can improve the translation efficiency of mRNA. Awe et al. [170] attempted to replicate Warren et al.'s [171] reprogramming method by transfecting synthetic OCT4 mRNA into human skin-derived primary cells (HUF1) using RNAiMAX as the transfection reagent. The total amount of synRNA and RNAiMAX used was 1.2 µg and 6 µl, respectively. The experimental results showed that the expression of OCT4 did not significantly increase statistically upon addition of B18R. Furthermore, no significant increase was observed even with higher amounts of synRNA, B18R, or their simultaneous use.

In summary, current studies show that B18R counteracts the IFN response and enhances transfection safety, but does not significantly enhance mRNA translation [172]. In some studies, B18R protein has been used as an adjuvant for mRNA translation: recombinant B18R protein (rB18R) was added as a soluble protein to cell culture medium, or B18R mRNA was co-transfected with mRNA encoding the target protein [173,174].

##### 2.1.1.3 E3 in the field of sa-RNAs translation

VACV E3 has been shown to be an efficient blocker of PKR activation and IFN-β up-regulation, and is therefore promising in the field of mRNA translation. *In vitro* experiments involving the transfection of GFP sa-RNAs into HFF cells resulted in robust phosphorylation of PKR and consecutive phosphorylation of eIF2α. To address this phenomenon, Beisser et al. [172] co-transfected mRNA encoding VACV E3 and observed that E3 prevented the phosphorylation of PKR and eIF2α, significantly reducing IFN-β induction by approximately 90%. Although there was a significant decrease in IFN expression, no improvement in cell viability was observed, possibly due to the secondary role of the IFN response in sa-RNAs-induced cell death. Subsequently, researchers tested the effect of E3 protein on the exogenous expression of the GOI. To assess the expression of GFP sa-RNAs in transfected HFF cells, researchers added mRNA encoding near-infrared fluorescent protein (iRFP) to each sample. In the absence of VACV proteins, approximately 8% of iRFP-positive cells expressed GFP at detectable levels. However, co-transfection with E3 mRNA tripled the sa-RNAs expression rate, leading to a 12-fold increase in the mean fluorescence intensity (MFI) of GFP. Moreover, further validation was performed using sa-RNAs encoding secreted version of a deep-sea shrimp luciferase (Nanoluc®), which showed a significantly increased accumulation of Nanoluc® by 35-fold following co-transfection with E3. These data suggest that E3 is not only effective in suppressing the innate immune response *in vitro*, but also enhances the expression level of sa-RNAs-encoded GOI.

*In vivo* experiments have also shown outstanding performance of the E3 protein. Xue et al. [175] explored its application in a diabetic ulcer model. They initially found that isomannide-derived LNPs (DIM1T LNPs) effectively delivered RNA to adipose stem cells (ASCs). Using DIM1T LNPs to co-deliver Firefly luciferase (FLuc) sa-RNAs and E3 mRNA complexes (SEC), the results indicated that the SEC significantly reduced the levels of phosphorylated PKR and eIF2α in ASCs, enhancing luciferase expression, which remained stable for at least 48 h post-treatment. DIM1T LNPs encapsulating SEC at a 0.5 mass ratio induced the highest luminescence intensity. In a diabetic skin wound model, DIM1T LNP-SEC engineered ASCs (DS-ASCs) prolonged the expression of hepatocyte growth factor (HGF) and C-X-C motif chemokine ligand 12 (CXCL12), demonstrating superior wound healing efficacy compared to wild-type (WT) and DIM1T LNP-mRNA counterparts. In summary, E3, whether co-delivered with sa-RNAs *in vitro* or in an *in vivo* model, can serve the dual purpose of reducing the level of innate immune response and enhancing the efficiency of sa-RNAs translation.

##### 2.1.1.4 Combined application of EKB

Application of B18R alone is not sufficient to enhance mRNA expression. It may be feasible to use a combination strategy with multiple immune evasion proteins.For instance, Poleganov et al. [168] developed an efficient method for generating induced pluripotent stem cells (iPSCs) through the co-transfection of a potent synthetic mRNA/miRNA cocktail. This cocktail promotes reprogramming by combining three VACV immune evasion proteins, E3, K3, and B18R (referred to as EKB), to enhance translation of mRNAs while mitigating cytotoxicity. Specifically, co-transfecting HFF with non-modified synthetic mRNAs encoding EKB and enhanced GFP (eGFP) demonstrated that co-transfection of E3 and K3 mRNAs reduced IFN-β transcript levels by 80% and tripled the translation of GFP mRNA. When B18R mRNA or rB18R was added, the translation increased by 4.6-fold, and IFN-β transcript levels further decreased. Furthermore, repeating the experiment with Luci mRNA, EKB mRNAs significantly increased translation, notably extending the duration of translation. After 72 h of transfection, Luciferase expression in the EKB samples was higher compared to the control group. Additionally, unprotected HFF subjected to four daily transfections with non-modified mRNA died, whereas co-transfection with EKB mRNAs rescued HFF survival, which is essential for successful reprogramming. This study significantly shortened the transfection cycle for generating iPSCs using RNA, demonstrating for the first time the establishment of a robust, integration-free reprogramming protocol for blood-derived iPSCs, avoiding invasive procedures such as skin punch biopsies. This represents a crucial step towards the clinical applicability of iPSCs.

Similarly, Beissert et al. [172] combined unmodified reprogramming sa-RNAs with EKB mRNAs to investigate whether the immune evasion protein mRNAs could block sa-RNAs-mediated PKR activation and IFN response. They transfected GFP sa-RNAs into HFF using electroporation or lipofection (1:4 (w/v) of RNA to RNAiMAX or MessengerMax solution; 5 µg/ml of final RNA concentration). The results showed that co-transfection with the three mRNAs significantly reduced IFN-β induction by 95% and generated a synergistic effect, yielding the highest frequency of GFP-positive cells, significantly higher than the control group without EKB transfection. Furthermore, the researchers tested whether this enhancement by immune evasion proteins could be reproduced *in vivo*. Using immunocompetent BALB/c mice, they injected RNA into the thigh muscle, either injecting FLuc sa-RNAs alone or co-injecting with EKB mRNA. In the control group mice, bioluminescence imaging detected almost no signal, whereas in the mice co-injected with EKB mRNAs, high-intensity luminescence signals were detected at the injection site, lasting for over two weeks. The experiments demonstrated that co-transfection with EKB mRNAs could mitigate the immunogenicity of synthetic mRNA and significantly enhance mRNA translation efficiency.

In summary, the B18R, E3 and K3 proteins derived from VACV can suppress relevant immune responses or enhance the efficiency of nucleic acid expression in the field of mRNA translation. The three also have synergistic effects, and the expression efficiency of mRNA will be greatly increased when delivered together.

#### 2.1.2 Influenza A Virus: NS1

Influenza A virus (IAV) is a highly contagious respiratory pathogen that causes seasonal epidemics. It is characterized by its segmented, negative-sense, ssRNA genome consisting of eight segments encoding at least 10 proteins, including hemagglutinin (HA), neuraminidase (NA), matrix proteins 1 (M1) and 2 (M2), polymerase acidic (PA), polymerase basic (PB), nucleoprotein (NP), and others. The IAV particles are enveloped by two abundant membrane proteins, HA and NA, as well as a small amount of M2 ion channel proteins. The M1 protein is located beneath the lipid membrane, providing structural support to the viral particles [176].Additionally, several viral proteins are not included in the IAV virion but are expressed only in infected host cells. These are termed non-structural proteins (NSPs), including NS1 and NS2. These proteins regulate viral replication and transcription, impacting the virus life cycle. More importantly, they also facilitate evasion of host defenses and viral pathogenicity by inducing apoptosis, interfering with innate immunity, and exacerbating inflammation [177,178].

The NS1 protein is composed of an RNA-binding domain that facilitates protein-RNA interactions and an effector domain that mediates protein-protein interactions [179,180]. There has been extensive research on the role of NS1 in mRNA translation. Phua et al. [181] found that NS1, when co-delivered in mRNA form, acts as a potent, non-toxic enhancer of mRNA transfection. They initially screened six different NS1 variants from different influenza virus subtypes through *in vitro* experiments using Stemfect mRNA transfection reagents to transfect BJ fibroblasts. The results of the experiments targeted NS1-TX91 (A/TX/36/1991 TX91; a pre-2009 human seasonal H1N1 virus). Cells co-transfected with NS1 mRNA significantly increased transfection efficiency, produced less IFN, and showed no significant difference in cell viability compared to controls, suggesting that NS1 is not toxic to fibroblasts. Furthermore, the *in vivo* efficacy of NS1-TX91 was investigated. They subcutaneously injected low doses of naked luciferase/NS1-TX91 mRNA (2 µg/2 µg) into the ear pinna and tail base of ICR mice. The results showed that the initial co-injection (luciferase/NS1-TX91 mRNA) did not significantly increase luciferase expression. However, when a second dose was co-injected at the same site 20 h later, mice receiving the combined injections miraculously expressed higher levels of luciferase compared to controls (luciferase/GFP mRNA). The difference in bioluminescence increased over time. Notably, the effect of NS1-TX91 became evident only after the second administration and exhibited sustained activity; in contrast, luciferase expression in the control group nearly ceased after 72 h, whereas the co-injection group maintained it for up to 120 h. To investigate the amino acid determinants underlying this enhanced transfection, the researchers conducted gain- and loss-of-function experiments with functional amino acids. They demonstrated that the transfection enhancement was mediated by NS1’s effector domain, which inhibited IRF3, PKR, and cleavage and polyadenylation factor subunit 30 kDa (CPSF30).

Additionally, the research team observed that the efficiency of mRNA transfection mediated by NS1-TX91 varied depending on mRNA modification and cell type. They transfected different types of cells with luciferase/mRNA complexes of NS1-TX91 in unmodified (UM), ψ-modified (single modification, SM), and ψ/m5C-modified (dual modification, DM) forms, and compared the expression of luciferase. The results indicated that unmodified mRNA outperformed modified mRNA in various cell types, including HepG2, RAW 264.7 and HeLa cells. Considering the higher costs and lower yields associated with the modification of mRNA during *in vitro* transcription, these findings suggest that the incorporation of NS1-encoded mRNA could be a pivotal strategy for reducing costs while enhancing the efficiency of mRNA technology.

Liu et al. [182] conducted similar studies using NS1 from another subtype, NS1-HK97 (A/Hong Kong/156/1997), across a wider range of cell types. Specifically, they used BJ fibroblasts (highly relevant for cellular reprogramming), HepG2 cells (the liver is an attractive target organ for non-viral gene therapy), RAW 264.7 cells (macrophages representing immune cells), primary mouse embryonic fibroblasts (pMEF, which have high biological relevance and *in vivo* application correlation), and Vero cells (used as controls because they do not produce IFN). These cells were seeded in 48-well plates and transfected using Stemfect transfection reagent with 0.25 µg/well of GFP mRNA and 0.25 µg/well of NS1 mRNA or luciferase mRNA (control). The results indicate that co-transfection of NS1 and GFP mRNA significantly enhanced GFP expression in all cell types capable of producing IFN. In contrast, this was not observed in IFN-deficient Vero cells, suggesting that the efficiency enhancement mediated by NS1 is IFN-dependent.

In terms of security, unlike NS1-TX91, which did not show a significant difference in viability compared to the control group, NS1-HK97 demonstrated better performance in terms of cell viability. A slight increase in survival rates was observed across all cell groups, indicating that NS1-HK97 can mitigate the toxicity associated with mRNA transfection. To understand the ability of NS1-HK97 to reduce mRNA-induced toxicity, they further studied the toxic effects of two different types of NS1: the first type, which does not inhibit CPSF30 (*e.g.*, NS1-HK97), and the second type, which inhibits CPSF30 (*e.g.*, NS1-TX91). CPSF30 inhibition is a unique immune evasion mechanism of IAV, which reduces the poly(A) tail length of endogenous pre-mRNA to suppress host gene expression, thereby preventing nuclear export and countering the host cell's innate immune response during early stages of viral infection. The results indicated that cells transfected with NS1-HK97 not only had the highest survival rates but also survived better than non-transfected cells. In contrast, NS1-TX91 was exceptionally effective in enhancing mRNA translation, though they negatively impacted cell viability. However, at normal transfection doses, this effect was considered tolerable (viability greater than 80%).

In further explorations, the roles of immune evasion proteins from VACV (EKB) and IAV (NS1) in enhancing mRNA translation was compared [183]. The experimental results indicated that NS1-TX91 exhibited a significantly higher enhancement level than VACV-EKB. Additionally, there was no synergistic effect observed between EKB and NS1-TX91, maybe on account of some overlap in the immune evasion mechanisms of E3 and NS1. Wang et al. [184] expanded application of NS1 to bone regeneration therapy. They employed Lipofectamine MessengerMax (LFM) transfection reagent to co-deliver NS1 mRNA and bone morphogenetic protein-2 (BMP-2) mRNA into mouse pluripotent stem cells (C3H10T1/2). When the mass ratio of BMP-2 mRNA to NS1 mRNA was 3:1, BMP-2 expression was the highest, increasing eightfold. This co-delivery also enhanced the expression of osteogenic markers, such as alkaline phosphatase, type I collagen, osteopontin, and osteocalcin, and boosted extracellular mineralization, thereby promoting osteogenic differentiation. This study demonstrated the potential of using NS1 mRNA in bone regeneration therapies, supporting the auxiliary role of NS1 in mRNA-based regenerative medicine.

#### 2.1.3 Middle East Respiratory Syndrome Coronavirus: ORF4a

Middle East Respiratory Syndrome (MERS) is a viral respiratory illness caused by the Middle East Respiratory Syndrome Coronavirus (MERS-CoV) [185,186]. MERS-CoV possesses a large RNA genome of ∼30 kb, containing at least 10 ORFs, and a variety of structural proteins. The *spike (S) protein* is a type I transmembrane glycoprotein expressed on the viral envelope surface, forming spikes that play crucial roles in virus binding, fusion, and entry into host cells. The *envelope (E) protein* is an integral membrane protein involved in intracellular transport, host recognition, virus assembly, and budding. The *membrane (M) protein* is essential for virus assembly, envelope formation, and interaction with the *nucleocapsid (N) protein* to form the viral core. The N protein, a phosphorylated basic protein, is the second largest structural protein of MERS-CoV, binding with the RNA genome to form the nucleocapsid, which is critical for viral replication and assembly. Additionally, MERS-CoV's genome encodes several accessory proteins (from dORFs: ORF3, ORF4a, ORF4b, ORF5) that can antagonize the host's antiviral response, typically the type I IFN response, contributing to the virus's pathogenicity and disease mechanism [187].

In the process of screening a library of sa-RNAs constructs encoding innate inhibiting proteins (IIPs), Blakney et al. [188] investigated the impact of these constructs on protein expression and immunogenicity. Experimental results revealed that among the constructs tested, the addition of the MERS-CoV ORF4a construct to the delivery of FLuc sa-RNAs using pABOL (a bioreducible polymer carrier) exhibited the most significant enhancement in protein expression, with a respective increase of 893-fold and 109-fold in FLuc expression observed in human cervical cancer cells (HeLa) and human fetal lung fibroblast cells (MRC5). Subsequently, *in vivo* validation was conducted in C57BL/6 mice (high producers of IFN-α/β and IFN-γ), which showed a slight increase in the total area under the curve (AUC) of FLuc protein expression over time upon the addition of MERS-CoV ORF4a construct sa-RNAs, indicating enhanced protein expression in cells capable of IFN sensing.

To further investigate the underlying mechanisms, researchers transfected WT and MERS-CoV ORF4a constructs into MRC5 cells and analyzed the levels of active NF-κB and IRF3 in nuclear extracts at 4-, 24- and 48-h post-transfection. The experimental results indicated that in samples treated with WT FLuc sa-RNAs or tumor necrosis factor-α (TNF-α) (as a positive control), active NF-κB was upregulated at 4 h, while the MERS-CoV ORF4a construct group significantly reduced the levels of active NF-κB. In human peripheral blood mononuclear cells (PBMCs), inflammatory factors such as IFN-α, IL-1β, IL-6, MIP-1β and MIP-3α were upregulated under the influence of sa-RNAs, while the construct group showed a slight but not significant downregulation of IL-1α, IL-2 and MIP-3α. The study demonstrates that the MERS-CoV ORF4a accessory protein interferes with the innate antiviral signaling pathways, including NF-κB-mediated responses, thereby enhancing expression efficiency during sa-RNAs translation.

#### 2.1.4 Parainfluenza Viruses: PIV-5 V

Parainfluenza viruses (PIVs) are single-stranded enveloped RNA viruses of the Paramyoviridaie family and are the second most common cause of severe acute respiratory illness in infants worldwide [189]. PIVs pose a significant threat to various vulnerable populations such as organ transplant recipients, immunocompromised individuals, chronically ill patients, and the elderly [190]. PIVs have a genome length 15–17 kb, encoding six major structural proteins, including nucleoprotein, phosphoprotein, matrix protein, fusion protein, hemagglutinin-neuraminidase protein, and RNA polymerase protein. Furthermore, the PIV genome encodes a multitude of proteins involved in viral replication [191,192].

PIV-5 V, similarly selected from an IIPs library, has been identified to enhance mRNA expression *in vitro* [188]. In both HeLa and MRC5 cells, delivery of Luci sa-RNAs using pABOL led to a respective increase of 796-fold and 72-fold in expression when the PIV-5 V construct was incorporated. Subsequently, the research team employed a more clinically relevant human skin explant model for *in vivo* validation, using EGFP sa-RNAs as the reporter gene. Doses of the WT construct was escalated from 0.2 µg to 2 µg, resulting in an increase in the percentage of EGFP-positive (+) cells from 10% to 18%. However, upon further increasing the dose to 20 µg, the percentage of EGFP+ cells plummeted to 5%, indicating a decrease in protein expression levels due to the escalation of nucleic acid dose. Conversely, for the PIV-5 V construct, there was a linear increase in the proportion of EGFP+ cells with increasing sa-RNAs doses. The proportion of EGFP+ cells reached 12% at a dose of 0.2 µg, increased further to 15% at 2 µg, and rose to 25% at 20 µg. These data suggest that PIV-5 V enhances expression in immune cells by increasing the percentage of cells expressing sa-RNAs.

Overall, these findings suggest that IIPs such as PIV-5 V and MERS-CoV ORF4 can be directly encoded into sa-RNAs vectors to modulate the activation of innate immune responses, thereby achieving high-level expression of target proteins and expanding the therapeutic window for RNA delivery.

#### 2.1.5 Other viral proteins to be explored and the related risks that need to be considered

Further identification and analysis of such viral proteins may lead to the discovery of more effective candidate drugs, providing valuable solutions for the development and application of mRNA therapies that are both highly effective and low in toxicity. For instance, Best et al. [193] initially demonstrated that Langat virus (LGTV) infection suppressed the expression of reporter genes driven by IFN-α/β- or IFN-γ-responsive promoters, indicating that LGTV is capable of inhibiting the IFN-mediated JAK-STAT signaling pathway. The researchers further expressed all the NSPs of LGTV individually and examined their ability to inhibit signal transduction to identify the viral proteins at work. The results indicated that the sole expression of LGTV-NS5 could suppress the response to IFN, thereby identifying NS5 as a potential IFN antagonist and, consequently, a potential mRNA translation enhancer.

Borna disease virus (BDV) is a non-segmented negative-sense RNA virus capable of establishing persistent infections, which is closely associated with its ability to suppress host IFN expression. Unterstab et al. [194] investigated this capability and found that the phosphoprotein (P protein) encoded by BDV can interact with TBK1 to inhibit IFN induction. Furthermore, the P protein can also be phosphorylated by TBK1, competing with IRF3, which acts as a kinase substrate. Based on this, the BDV-P protein is worthy of investigation in the field of mRNA translation. It has been discovered that human metapneumovirus (HMPV) can suppress TLR4-mediated NF-κB activation, RIG-1-mediated IRF-3 activation, and Type I IFNR-mediated JAK-STAT pathway activation. Regarding its specific mechanism, the G protein derived from HMPV inhibits the activation of host cell TLR4 and the recognition of the viral genome by RIG-1. Therefore, the HMPV-G protein may also facilitate mRNA translation by suppressing the immune response induced by mRNA [145].

In summary, the use of viral proteins as mRNA translation boosters shows promise, yet it is accompanied by a series of potential risks that cannot be overlooked. It is essential to weigh the benefits of enhanced expression efficiency against these potential risks and conduct a careful evaluation.For instance, there is the risk of off-target immunomodulation. Viral proteins, as foreign antigens, may be recognized by the host immune system, triggering specific antibody or T cell responses. If these proteins possess cross-reactive epitopes with host proteins, it may give rise to autoimmune reactions (such as through the molecular mimicry mechanism), leading to an attack on normal tissues [195]. Certain viral proteins (such as specific domains of the spike protein) may activate the innate immune system via PRRs (such as TLRs), resulting in excessive inflammatory responses (such as elevated levels of IL-6 and TNF-α), and triggering systemic inflammation or local tissue damage. Some viral proteins (such as the immune evasion proteins of poxviruses or herpesviruses) may interfere with the host immune signaling pathways (such as the IFN pathway). Prolonged expression may suppress antiviral immunity and increase the risk of secondary infections. In addition, there are also risks associated with genomic integration. If the used viral proteins are derived from retroviruses (such as the HIV integrase) or have DNA binding/cutting functions (such as the adeno-associated virus Rep protein), they may mediate the integration of exogenous mRNA or vector DNA into the host genome, leading to insertional mutations, activation of proto-oncogenes, or inactivation of tumor suppressor genes [196,197].

In response to these risks, some strategies can be considered. Protein engineering modification can be carried out. By deleting immunodominant epitopes, immunogenicity and cellular damage can be reduced [198]. Tissue-specific vectors (such as targeted modification of LNPs) can be adopted, or local administration can be used to reduce off-target exposure. Alternatively, without using the viral genome, synthetic proteins that mimic the immune evasion mechanisms of viruses can be employed. For instance, Zheng et al. [199] discovered that the main protease NSP5 of SARS-CoV-2 inhibits the expression of IFN-related genes by cleaving the host translation initiation factor eIF4G. Inspired by this, researchers designed an artificial protein that selectively inhibits excessive immune responses by targeting specific domains of eIF4G while preserving the key pathways of antiviral defense [[199], [200], [201]].

### 2.2 Short artificial RNA molecules

RNA interference (RNAi) is a process that effectively silences or inhibits the expression of target genes by selectively inactivating the corresponding mRNA of the target gene through dsRNA. This process can be induced by small interfering RNA (siRNA) or short hairpin RNA (shRNA) to degrade the target mRNA [[202], [203], [204]].

**siRNA:** Angel et al. [205] conducted a combinatorial screening effort to identify siRNA mixtures that could desensitize cells to exogenous RNA. The results showed that a combination of IFN-β, PKR and STAT2 knockouts rescued cells from the innate immune response triggered by frequent long RNA transfections, was able to achieve transfection of primary human fibroblasts with RNA encoding the reprogrammed proteins OCT4, SOX2, KLF4 and undifferentiated embryonic cell transcription factor (UTF1) every 24 h. Furthermore, these transfected cells exhibited sustained expression and activity of the encoded proteins over multiple days. This approach targeting various immune-associated molecules and suppressing multiple factors underscores the imperative for effective circumvention of the immune response.

**shRNA:** In the generation of iPSCs by retroviral overexpression of reprogramming factors (OCT4, SOX2, KLF4, c-MYC), Lee et al. [206] utilized shRNA to inhibit the function of TLR3, TRIF, and MyD88, an approach that can also be applied to inhibit the immunogenic response during mRNA translation, thereby improving the efficiency .

**miRNA:** However, theoretically, siRNA and shRNA can also trigger innate immunity by inducing Type I IFN signaling through TLR3, which seems contradictory to their use in enhancing mRNA transfection efficiency and may potentially diminish their effectiveness in this regard to some extent. Therefore, microRNA (miRNA) may be considered a more favorable alternative due to its inherent ability to regulate inflammatory responses, thereby reducing the likelihood of immune activation [207,208].Studies by Grogg et al. [209] investigated the changes in miRNA expression following exposure to metal nanoparticles (NPs) in a mouse neuronal co-culture model. The results showed that exposure to manganese (Mn) NP resulted in oxidative stress, inflammation, and toxicity. Next-generation sequencing (NGS) identified several potential miRNA candidates involved in regulating NP-induced responses. Among these candidates, miR-155 emerged as the primary candidate, and reintroduction of miR-155 mimics into co-cultures effectively suppressed gene and protein expression of inflammatory markers TNF-α and IL-6 induced by Mn NPs. Although this study did not involve the transfection of IVT mRNA, the function of miRNA demonstrated in reducing innate immune responses may warrant further investigation in the field of mRNA translation.

**ds-cRNA:** Circ-RNAs have higher stability, unique folding properties, and lower immunogenicity than linear RNAs, making them potential candidates for novel biomedical applications, such as remodeling into new RNA aptamers and protein translation vectors [47,[210], [211], [212], [213]]. Chen et al. [214] synthesized low-immunogenic circ-RNA aptamers with molecularly internal short double-stranded structures (ds-cRNA) by optimizing RNA self-splicing to form circles. They demonstrated the conformational stability of circ-RNA aptamers, their ability to inhibit PKR activation, and the mechanism of inhibition at the single-molecule level. This study established a mouse model overexpressing ds-cRNA for the first time, proving the safety of circ-RNA *in vivo*. Furthermore, in a mouse model of psoriasis, a disease related to PKR overactivation and inflammation, circ-RNA aptamers were targeted to the spleen and successfully intervened in the treatment of psoriasis. These findings laid a theoretical and technical foundation for the development of new drugs based on circ-RNA, and their ability to inhibit PKR activation also provides new possibilities for mRNA translation applications.

## 3 Non-viral small molecules, short peptides and proteins

**Fig. 3:**
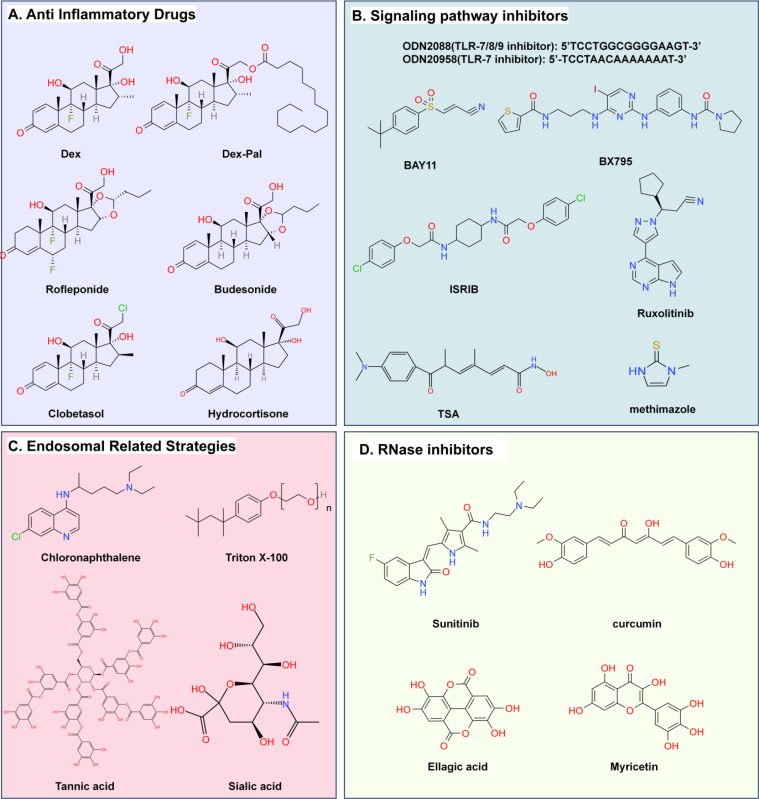
Representative structural diagrams of non-viral small molecules, short peptides and proteins.

The strategy of using large molecular drugs, such as natural viral proteins, to assist mRNA translation has been proven to suppress multiple key steps in the Type I IFN signaling pathway and may potentially enhance the efficiency of mRNA translation. However, in practical applications, the dissemination of macromolecular drugs faces certain challenges, such as the separation and purification of proteins and nucleic acids during the production process, and the stringent preservation conditions required during transportation, all of which led to increased costs. In comparison, small molecule drugs may possess greater market potential in these respects (Fig. 3).

### 3.1 Anti-inflammatory drugs

Exogenous mRNA and its delivery system have the potential to induce inflammation, significantly affecting the stability and translational efficiency of mRNA. Studies indicate that pretreatment with anti-inflammatory drugs, such as dexamethasone (Dex), before intravenous administration of mRNA LNPs, effectively controls adverse reactions [215]. In clinical trials for patisiran (Onpattro), the combined use of Dex, acetaminophen, and histamine H1 and H2 receptor antagonists was found to alleviate inflammation and enhance the efficacy of mRNA translation [216]. Therefore, the concurrent use of anti-inflammatory drugs during mRNA translation is a strategy that can be considered to suppress inflammation-induced immune responses and to enhance transfection efficiency.

#### 3.1.1 Intravenous administration

**Dexamethasone:** Dex is a potent synthetic corticosteroid primarily used for the treatment of various inflammatory conditions, autoimmune diseases, and certain hematological malignancies [[217], [218], [219], [220], [221]]. In addition to conventional therapy, it can also be used prophylactically to reduce inflammatory responses during antibiotic and certain chemotherapy treatments [222]. Similarly, Dex also serves as a valuable tool for mitigating inflammation associated with nucleic acid cargo delivery processes.The initially applied form is Dexe in its unmodified, pristine state. Ohto et al. [223] investigated the *in vitro* effects of Dex on the transfection activity of LNPs composed of ionizable lipids and IVT mRNA. Using LNPs containing ssPalmO-P4C2 (an auxiliary lipid), they transfected mouse MEF cells with luciferase as a reporter gene. They found that Dex primarily increased transfection activity during the mid to late stages (4–48 h) post-transfection. The overall luciferase activity indicated that Dex ultimately enhanced transfection activity by 1.6-fold. However, in this process, Dex still possesses certain limitations. Due to Dex's low hydrophobicity, the drug molecules tend to dissociate from the LNPs in the bloodstream, leading to systemic exposure to corticosteroids. This systemic exposure poses risks such as diabetes, hypertension, cataracts, and fractures. Therefore, increasing the hydrophobicity of the drug to achieve precise co-delivery of mRNA and the drug is highly necessary.

**Dex-Pal:** To achieve this objective, the aforementioned group further developed Dex palmitate (Dex-Pal) by conjugating Dex with lipids, thereby increasing its hydrophobicity. This approach enabled the co-encapsulation of the drug and Luci mRNA into LNPs. Intravenous injection of 0.25 mg/kg mRNA and 0.65 mg/kg Dex resulted in significantly enhanced protein expression in the mouse liver compared to the control group (without Dex-Pal). Specifically, protein expression increased by 2.7-fold in BALB/c mice and by 6.6-fold in C57BL/6 J mice (*P* < 0.05) [223].

**LD003:** Chen et al. [224] conducted a similar study in which Dex was chemically modified to be more effective and easily integrated into the LNP system. They utilized a biodegradable succinic acid linker to attach the hydrocarbon moiety to the hydroxyl group at the C21 position of Dex. These derivatives, composed of one or two hydrocarbon chains and an ionizable tertiary amine, were synthesized to mimic the amine lipids used for nucleic acid encapsulation in LNPs. Ultimately, five hydrophobic Dex derivatives (LD001–005) were developed to replace the amine lipids in the LNP formulation, thereby achieving a dual function of immune suppression and nucleic acid loading.To evaluate the effectiveness of these Dex derivatives in preventing immune activation induced by mRNA-LNP, FLuc mRNA, a reporter gene, was encapsulated in LNPs containing 0 or 10 mol% LD003. The LNPs were then subcutaneously injected into the interscapular region of C57BL/6 mice at a dose of 3 mg/kg. Blood samples were collected 2 h after the injection to detect the levels of plasma cytokines. By comparing the immunogenicity suppression effects between the group with LD003 and the group without LD003, it was found that the production of cytokines (IL-6, TNF-α, IL-12p70, IL-1β, IL-10) in the mRNA-LNP group containing LD003 was significantly lower than that in the mRNA-LNP group without the prodrug, almost returning to the baseline level. This method demonstrated a stronger immunosuppressive effect than the high-dose (20 mg/kg) free Dex. Moreover, due to the extremely low dosage of the prodrug formulation, it avoided the adverse reactions associated with high-dose corticosteroids. This study provides a convenient strategy for alleviating immune stimulation responses in gene therapy formulations administered systemically [224].

**Roflumilast and budesonide:** In addition to Dex, there are other anti-inflammatory drugs with similar applications. For instance, Davies et al. [225] synthesized prodrugs of roflumilast and budesonide with varying alkyl chain lengths to explore the relationship between the inflammatory response, protein synthesis, and systemic steroid exposure induced by their co-delivery with mRNA LNPs. In the experimental protocol section, they utilized chemically modified mRNA encoding the secreted protein fibroblast growth factor 21 (hFGF21 mRNA) and formulated it into LNPs using DLin-MC3-DMA (MC3) as an ionizable amine lipid. The MC3 LNP-formulated hFGF21 mRNA was administered intravenously to CD1 mice at a dose of 0.3 mg/kg mRNA. The experimental results indicated that when the parent steroids were incorporated into the LNP formulation without any modification, they rapidly entered the systemic circulation from the site of administration and were quickly eliminated, providing only limited protection and accompanied by a significant inflammatory response. However, when roflumilast and budesonide were incorporated into the LNP formulation as ester prodrugs, the appearance of the steroids in the plasma was delayed. Notably, the modification with longer alkyl chains can further delay and reduce the C_max_, extend the half-life, and permit sustained protein production levels and enhanced overall systemic protein exposure. Thus, the dosing regimen of this study is highly suitable for conditions requiring systemic exposure to therapeutic proteins, chronic treatments, and self-administration [225].

**Incorporating Dex into the LNP formulation:** Despite the superior performance of the aforementioned prodrugs, Zhang et al. [226] argue that, from a translational perspective, incorporating the unmodified form of Dex into LNPs might face fewer regulatory hurdles and scaling challenges compared to Dex prodrugs, potentially allowing for broader application of new LNP formulations. So, the research team leveraged the structural similarity between Dex and cholesterol to partially replace cholesterol with Dex in the development of LNP formulation recipes, aiming to reduce the induced inflammatory response.In *ex vivo* experiments using HepG2 cells treated with LNPs encapsulating Fluc mRNA, they found that C9D1 LNP (where the C:D ratio represents the weight ratio of cholesterol to Dex) had similar transfection efficiency to C10D0 LNP without increased cytotoxicity. However, as the proportion of Dex increased, transfection efficiency significantly decreased, even though LNPs could still be formulated. To verify whether Dex incorporation could suppress the immune response triggered by the LNPs themselves, the anti-inflammatory effect of C10D0 LNP and C9D1 LNP on mouse macrophages (RAW246.7) was assessed. After 24 h of LNP stimulation, the concentration of TNF-α in the supernatant indicated that C9D1 LNP treatment reduced the increase in TNF-α levels by half. This excellent anti-inflammatory effect was also effective *in vivo*. Studies on the inflammatory response using C57BL/6 mice showed that serum TNF-α concentrations in mice treated with C9D1 LNP were significantly lower than in those treated with C10D0 LNP, successfully mitigating the inflammatory response induced by LNPs. Furthermore, the transfection efficiency was increased by 1.5-fold [226]. In summary, directly incorporating Dex into the mRNA LNP formulation is also a strategy that can be considered. This approach can serve to alleviate immune responses and enhance transfection efficiency, and it is more convenient to use compared to prodrugs that require certain chemical modifications.

#### 3.1.2 Local topical administration

The aforementioned drugs are administered systemically via intravenous injection. In addition to this, the application of corticosteroids topically, such as in the form of ointments, is also a viable option to consider. This method is non-invasive, has a higher patient compliance rate, and is more readily accepted by patients [227,228]. Building on this, Zhong et al. [229] conducted research and found that the topical application of clobetasol propionate and hydrocortisone creams at the injection site could effectively suppress the type I IFN response induced by the ZIKVac-sa-RNAs vaccine. Specifically, hydrocortisone improved *in vivo* translation only on Day 2 and 3 post-injection, while clobetasol, by extending the translation period to a week, performed better, increasing the overall translation of mRNA by 3.5 times. Furthermore, the combined use of clobetasol with the NF-κB inhibitor BAY11 could further reduce the level of immune response. However, the application environment of such strategies requires certain attention, as clobetasol can prevent the formation of antibodies against the sa-RNAs-encoded antigens, making it unsuitable for use in vaccine administration environments, while it is likely to be beneficial for non-immune therapies that require avoidance of immune activation.

### 3.2 Signaling pathway inhibitors

The aforementioned glucocorticosteroids, such as Dex, exert direct anti-inflammatory effects by mechanisms such as inhibiting the aggregation of inflammatory cells and controlling the release of inflammatory mediators, thereby weakening and blocking the inflammatory response induced by mRNA LNPs, akin to extinguishing a flame with water. mRNA, as a PAMP, upon entry into cells, is recognized by PRRs, triggering a cascade of innate immune responses. In theory, blocking any link in this series of signal pathways may prevent the ultimate degradation of mRNA and termination of translation. Based on this, a variety of small molecule drugs can be considered for application in the field of mRNA translation. They target various processes of the immune response triggered by mRNA LNPs, from receptor sensing to signal transduction to cascade amplification, reducing and eliminating elements that fuel the fire, thus controlling the source and process of the inflammatory response. Next, this article will introduce, in the order of the immune response processes mentioned in the introduction, the inhibitors or antagonists of receptors or TFs that are currently being studied for application in the field of mRNA translation.

#### 3.2.1 TLR antagonists: ODN20958, ODN2088

When mRNA LNPs are taken up by cells, they are transported into endosomes. In the acidic environment of the endosome, the ionizable cationic lipid components of the LNP become protonated, disrupting the endosomal membrane and facilitating the escape of mRNA into the cytoplasm. During this process, if the mRNA molecule contains dsRNA, or if its sequence or modification has a specific structure, it may be recognized by TLR3, TLR7 or TLR8, triggering downstream immune responses. ODN20958 and ODN2088 are two antagonists of TLRs. They are designed with specific sequences that can block the binding of TLR7 and TLR9 to their ligands, thereby inhibiting TLR-mediated signal transduction and inflammatory responses, and suppressing the release of IFN-α and IL-6 [230], demonstrating the ability to modulate immune responses. ODN20958 (sequence: TCCTAACAAAAAAAT) is a TLR7 inhibitor, while ODN2088 (sequence: TCCTGGCGGGGAAGT) is an antagonist specifically targeting TLR9. It works by mimicking specific sequences found in mammalian DNA. In preclinical studies, ODN2088 has shown protective effects against liver inflammation and fibrosis.

Based on this, Zhong et al. [229] attempted to apply these DNA inhibitors in the field of mRNA translation. They investigated the ability of the water-soluble oligonucleotide ODN2088 and ODN20958 to suppress sa-RNAs-induced type I IFN responses *in vivo*. The experimental protocol involved co-administration of ODN2088 and ODN20958 with the ZIKVac-sa-RNAs vaccine in IFN-β luciferase reporter mice. The results indicated that co-administration of these two TLR inhibitors with the ZIKVac-sa-RNAs vaccine significantly reduced type I IFN responses immediately. However, this immunomodulatory effect disappeared after 1 d. Subsequently, they tried to enhance and prolong this regulatory capability by pretreating the injection site with ODN2088. The results showed that pretreating the injection site with ODN2088, and then administering these inhibitors twice daily via intradermal injection following ZIKVac-sa-RNAs injection, only slightly increased and prolonged the suppression of the IFN-β response. In summary, ODN2088’s ability to significantly reduce innate immune responses is limited to immediate use following mRNA administration. Building on this, in terms of the *in vivo* translational efficiency of sa-RNAs, when using sa-RNAs encoding luciferase (LUC-sa-RNAs) administered alone via intradermal electroporation, ODN2088 failed to improve translational outcomes. Overall, the effectiveness of these DNA inhibitors appears to be relatively limited. Nonetheless, ODN2088 is a good choice for combination therapy, as better therapeutic effects were observed when mRNA was used in conjunction with chloroquine and ODN2088.

#### 3.2.2 TBK1 and IKKɛ antagonist: BX795

As key components of signal transduction, TBK1 and IκB kinase epsilon (IKKɛ) are involved in the signaling pathways mediated by various PRRs. They activate downstream IRFs, thereby promoting the production of type I IFNs and inflammatory cytokines. BX795 is a well-known small molecule inhibitor of TBK1 and IKKɛ. By inhibiting the activity of these two kinases, BX795 can reduce signal transduction mediated by TBK1 and IKKε, suppress the phosphorylation of IRF3 [101], and thereby suppress inflammatory responses and the production of type I IFNs, ultimately inhibiting a multitude of cytokines, enzymes, and chemokines associated with mRNA degradation [231]. Awe et al. [170] explored the application of BX795 in the field of mRNA translation, investigating whether BX795 could effectively block immune responses to synthetic RNA and stabilize the expression of OCT4 in HUF1 cells. Results showed that when combined with 120 ng/µl of total synRNA, a low concentration (0.001 µM) of BX795 induced widespread and uniform expression of OCT4. However, this effect did not intensify with increasing doses. Subsequent studies increased the concentration of BX795 to 1 µM, resulting in more cells expressing OCT4, but overall expression levels were comparable to those at 0.001 µM concentration of BX795. However, compared with the control group, cell proliferation assays revealed significant deficiencies in cellular proliferative activity with BX795. In summary, as an effective antagonist of TBK1 and IKKɛ, the clinical potential and safety of BX795 require further research and validation.

#### 3.2.3 NF-κB antagonist: BAY11

After undergoing complex signal transduction, the invasion of mRNA LNP within the cell leads to the production of a series of TFs, such as NF-κB, IRF3 and IRF7. NF-κB is a crucial TF that plays a key role in regulating various biological processes, including immune responses, inflammatory responses, cell growth, differentiation, and apoptosis. NF-κB is typically found in the cytoplasm, associated with the inhibitory protein IκB, forming an inactive complex. Upon cellular stimulation by external factors such as inflammatory cytokines, stress signals, or pathogen invasion, IκB is phosphorylated and degraded, releasing NF-κB, which then translocates to the nucleus to activate the transcription of target genes [[232], [233], [234], [235], [236]]. BAY11 is a well-documented inhibitor of the NF-κB signaling pathway; it suppresses the activity of IκB kinase (IKK), preventing the phosphorylation and degradation of IκB, thereby inhibiting the activation and nuclear translocation of NF-κB. Experimental studies have shown that BAY11 not only inhibits the activity of NF-κB but also reduces the production of inflammatory factors. It has inhibitory effects on multiple cytokines and signaling pathways, including the suppression of extracellular signal-regulated kinase (ERK), p38, and TBK1 phosphorylation or activation, leading to the inhibition of activator protein-1 (AP-1), IRF-3, and STAT-1 translocation and activation. Therefore, BAY11 possesses broad anti-inflammatory effects and holds potential to assist in the translation of mRNA, enhancing its safety and improving transfection efficiency.

Awe et al. [170] first discovered and demonstrated that using BAY11 can augment mRNA translation efficiency by inhibiting NF-κB. Specifically, in their experiment, synthesized OCT4 mRNA was transfected into HUF1 cells using RNAiMAX transfection reagent, followed by continuous replenishment of fresh culture medium and BAY11 for a duration of 5 d. Immunocytochemical analysis was employed to quantify OCT4 expression, revealing that BAY11 at low concentration (0.01 µM) resulted in robust OCT4 stability and more uniform expression, while the highest level of OCT4 expression was observed at a high concentration (1 µM). Additionally, cell proliferation assays were conducted on HUF1 cells, which showed a significant impairment in cell proliferation with 1 µM of BAY11 compared to the control group. In brief, the concentration of 0.01 µM BAY11 achieved an optimal balance between robust OCT4 expression and statistically insignificant reduction in cell proliferation. Building on this foundation, Zhong et al. [229] attempted *in vivo* experiments in mice. They assessed the ability of BAY11 to inhibit the *in vivo* type I IFN response induced by ZIKVac-sa-RNAs. The results indicated that the effectiveness of BAY11 is highly dependent on the dosage and timing of administration. In terms of dosage, co-administration of ZIKV-sa-RNAs with BAY11 (25 µg per mouse) significantly suppressed the IFN-β response within 24 h, while co-administration with a lower dose of BAY11 (12.5 µg per mouse) was ineffective. Regarding the timing of administration, BAY11 was most effective when used immediately after mRNA administration, resulting in a significant reduction in innate immune responses; in contrast, pre-treating the injection site with BAY11 was ineffective. Furthermore, the researchers explored the possibility of combination therapy and found that the use of BAY11 in conjunction with betamethasone valerate exhibited a more pronounced reduction in type I IFN responses.

#### 3.2.4 IRF7 inhibitors: TSA

However, not all inhibitors that can suppress related receptors or factors can exert synergistic effects in the field of mRNA LNP, as shown in the following example. IRF7 is a key TF considered to be the primary factor in producing IFN-I and regulating innate immune responses. It works by binding to viral response elements, along with other TFs such as NF-κB and IRF3, thereby inducing the production of IFN-I.Trichostatin A (TSA) is a known inhibitor of IRF7, which reduces the production of IFN-I by inhibiting the activity of IRF7. The mechanism of action of TSA may involve the inhibition of histone deacetylase (HDAC) activity, affecting the function of various TFs, including IRF7, and thus influencing gene expression [[237], [238], [239]]. Therefore, TSA has potential application value in the regulation of immune responses. In the study by Drews et al. [169], using GFP mRNA as the reporter gene, during the transfection process of HFF1 cells with LF, attempts were made to apply TSA to inhibit the innate immune response triggered by exogenous mRNA. However, no significant downregulation of evaluated immune response-related genes was observed. Just as blocking a river may cause water to flow into other rivers that lead to the sea, this could be because even if IRF7 is inhibited, there are other compensatory pathways within the complex immune response that can take over, such as IRF3.

#### 3.2.5 JAK pathway inhibitors: Ruxolitinib

After the above-mentioned TFs enter the nucleus, they regulate the genome to produce a series of pro-inflammatory cytokines and type I IFNs. The secreted type I IFNs bind to IFN receptors through an autocrine/paracrine mechanism, activating the JAK signaling pathway and inducing the expression of PKR through the JAK-STAT pathway. The activated PKR phosphorylates the translation initiation factor eIF2α, leading to global translation inhibition (such as the shutdown of cap-dependent translation). Therefore, the use of JAK inhibitors can block the IFN signaling, reduce the activation of PKR, maintain the non-phosphorylated state of eIF2α, and thus protect the function of the translation initiation complex [[240], [241], [242], [243]].Ruxolitinib is a JAK pathway inhibitor that reduces the production of inflammatory cytokines by blocking signal transduction between JAK and STAT [244], showing certain efficacy in the treatment of certain inflammatory diseases and immune-mediated diseases. In 2011, Ruxolitinib was approved by the FDA for the treatment of myelofibrosis, highlighting its potential clinical utility [245]. Blakney et al. [188] applied ruxolitinib in the field of mRNA translation, conducting a challenge of Ruxolitinib in combination with MERS-CoV ORF4a constructs. They intravenously injected 5 µg Luc sa-RNAs into BABL/c mice, with experimental schemes divided into groups with or without the viral innate inhibitory protein MERS-CoV ORF4a, and with or without co-formulation with 100 µg ruxolitinib. Protein expression was quantitatively assessed at 4-, 7-, 10- and 14-d post-injection. At Day 4, the protein expression levels of the two formulations containing ruxolitinib were slightly higher compared to the WT or MERS-CoV ORF4a constructs; on Day 7, the protein expression levels of the two Ruxolitinib-containing formulations were higher compared to the sa-RNAs-only parallel group; by 14 d, no protein expression was observed in the sa-RNAs group without Ruxolitinib, while a small number of positive samples were still observable in the ruxolitinib-treated group. These data suggest that Ruxolitinib can moderately increase sa-RNAs protein expression and has a longer time dimension.

#### 3.2.6 Integrated stress response inhibitor: ISRIB

Upon entry of the aforementioned STAT factors into the cell nucleus, they regulate the genome to enter the so-called "integrated stress response (ISR)" state. ISR is an intracellular mechanism that is activated when cells face various stress conditions, such as nutrient deficiency, oxidative stress, endoplasmic reticulum stress, or viral infections. At the initial stage of ISR activation, specific kinases within the cell, such as PKR, are activated. These kinases can sense the cellular stress state and promote the phosphorylation of the eIF2. When eIF2 is phosphorylated, it binds to eIF2B, thereby inhibiting the activity of eIF2B and ultimately slowing down protein synthesis. This slows the overall translation process of the cell, allowing the cell to focus resources on responding to stress, and consequently significantly reducing the transfection efficiency of mRNA LNP [[246], [247], [248], [249]].

ISRIB (Integrated Stress Response Inhibitor) is a small molecule inhibitor that specifically inhibits the ISR. During cellular stress responses, ISRIB can prevent the transition of eIF2B to an inhibitory conformation and promote the formation of an active conformation, thereby suppressing the activation of the ISR pathway. Studies have shown that the development of various neurological disorders is associated with the persistent activation of ISR, and the inhibitory effect of ISRIB helps protect neurons from stress-induced damage. It is currently primarily used in research on neurological disorders [[250], [251], [252]]. In addition, ISRIB may also treat diseases related to protein homeostasis imbalances, such as applications in the field of mRNA, where it can suppress immune stress and restore translation efficiency.Ohto et al. [223] explored this area by examining the effect of ISRIB on the efficiency of *in vitro* transfection of mRNA LNP. The results showed that when LNPssPalm was used as the transfection vector and Luci mRNA as the reporter gene, ISRIB mainly enhanced the transfection activity in the early stage (0–6 h). The early accumulation of luciferase activity indicated that ISRIB increased transfection activity by 1.4-fold. This suggests that ISRIB has potential in synergizing with mRNA translation.

#### 3.2.7 INF inhibitors

In addition to the aforementioned signaling pathway-related inhibitors, there are also many small molecule IFN inhibitors available on the market that could theoretically assist in mRNA translation. Liu et al. [253] conducted exploratory research on this topic. They screened a series of commercially available small molecule IFN inhibitors. Prior to transfection, BJ fibroblasts were pre-treated with the drugs for 5 h, then GFP mRNA was transfected using the Stemfect Transfection Kit to assess the potential of the drugs to enhance mRNA transfection. The experimental results indicated that within the non-toxic range, none of the tested small molecules could enhance transfection, and even one-third of the tested compounds inhibited GFP expression. Specifically, for most cardiac glycosides (IFN inhibitors), significant inhibition of GFP mRNA expression was observed; for natural compounds inhibiting NF-κB (digoxin, ursolic acid, berberine, and quercetin), no enhancement of GFP expression was observed even at higher concentrations; for TLR3 inhibitors (sertraline, resveratrol, flufenamic acid, amlodipine besylate and trifluoperazine), a statistically significant inhibition of IFN production was observed at higher concentrations for all drugs except amlodipine besylate, but no enhancement of GFP expression was observed for any drug; for PKR inhibitors (C16 and 7DG), both effectively inhibited IFN production, however, no enhancement of GFP expression was observed. Although the above results are negative, it does not mean that these drugs are completely hopeless in assisting mRNA translation, as the drug administration time in this study was 5 h prior to administration, without investigating the situation of simultaneous or delayed drug administration, thus further exploration is needed in this area.

### 3.3 Endosome-related strategies

The aforementioned drugs are designed to suppress immune responses, thereby reducing the impact of IFNs on mRNA and enhancing transfection efficiency. In addition to this, there are numerous factors that affect the stability of mRNA after LNPs enter cells, with endosomal degradation being the most significant.Endosomal recycling refers to the process by which extracellular macromolecules, ligand/receptor complexes, functional membrane proteins, and lipids are internalized into cells through endocytosis. Subsequently, they undergo a series of sorting and directed transport processes within the endosomal structure. Some macromolecules are returned to the plasma membrane through endocytic recycling, while others are transported to the Golgi apparatus via retrograde transport, and the remainder are delivered to lysosomes by late endosomes for degradation [254].Currently, there are two primary strategies to enhance mRNA transfection efficiency from the perspective of endosomal processes. One is endosomal escape, which facilitates the egress of mRNA that has already entered the endosome into the cytoplasm. The other is the inhibition of endosomal recycling, which reduces the quantity of mRNA NPs transported to lysosomes and increases their concentration in the cytoplasm, thereby improving the translational efficiency of mRNA.

#### 3.3.1 Endosomal escape accelerator

Naked mRNA (mRNA that is not encapsulated in a delivery vector) is primarily endocytosed through scavenger receptors on the cell membrane. These receptors are widely distributed in macrophages, dendritic cells, hepatocytes, etc., and are mainly responsible for recognizing negatively charged macromolecules (such as free nucleic acids, oxidized lipoproteins, etc.) [255,256]. After naked mRNA binds to the receptors, the cell membrane invaginates to form an endosome. Subsequently, the endosome fuses with the lysosome, leading to the exposure of mRNA to the acidic environment of the lysosome and nucleases (such as DNase II and RNase). Some endosomes may rupture before maturing into lysosomes, randomly releasing a small amount of mRNA. However, most naked mRNA cannot actively disrupt the membrane of the endosome and is degraded in the lysosome. Only less than 1% of the mRNA can escape into the cytoplasm and be translated into proteins by the host ribosomes, and the type of the protein is completely determined by the coding sequence of the mRNA. Therefore, cationic lipids and polymeric NPs are widely used for mRNA delivery, utilizing various endocytic pathways (such as lectin-dependent endocytosis and clathrin-dependent endocytosis) to enter cells [[257], [258], [259], [260]]. Taking cationic liposomes as an example, when mRNA LNPs are taken up by cells, they are transported to endosomes. In the acidic environment of the endosome, the ionizable cationic lipid components of the LNP are protonated, which disrupts the endosomal membrane and facilitates the escape of mRNA into the cytoplasm. Additionally, membrane fusion techniques [[261], [262], [263]], such as Entos's Fusogenix nucleic acid delivery technology, utilize proprietary Fusogenix protein-lipid vectors (PLVs) for drug delivery. Fusogenix PLVs, composed of fusion-associated small transmembrane proteins (FAST), can directly deliver mRNA or DNA to target cells without the need for endocytosis-mediated pathways. However, overall, the proportion of mRNA NPs that can escape from endosomes and be translated is still low, with most being delivered to late endosomes or lysosomes, leading to mRNA degradation [264,265]. Therefore, finding methods to promote endosomal escape would be beneficial for both naked and NP-encapsulated mRNA translation.

##### 3.3.1.1 Chloroquine

Chloroquine (CQ) has long been recognized as an antimalarial drug. However, it also possesses superior anti-inflammatory and anticancer properties, and has been utilized in the treatment of various other conditions, such as systemic lupus erythematosus, rheumatoid arthritis, and liver abscesses caused by amoebiasis [[266], [267], [268], [269], [270]]. Furthermore, in the realm of mRNA translation, CQ has garnered extensive attention due to its capability to enhance endosomal escape [271]. This capability can be elucidated from both chemical and osmotic pressure perspectives. From a chemical perspective, CQ is a weak base that readily enters cells and becomes protonated, sequestering protons within the endosome. After accumulating in the acidic endosome, CQ raises the pH of the internal environment, thereby inhibiting the digestive activity of lysosomal enzymes. As the alkalinity increases, the activity of P-glycoprotein (P-gp) present in the lysosomal membrane is significantly reduced, thereby enhancing the flux of drugs from the internal environment to the cytoplasm [272].From the perspective of osmotic pressure, this phenomenon can also be reasonably explained [272]. At neutral pH, CQ exists in its non-protonated form, making it relatively lipophilic and easily able to penetrate cells and subsequently reside within endosomes. However, when exposed to the acidic environment of endosomes, CQ converts to its protonated form, becoming too hydrophilic to rapidly diffuse across the endosomal membrane, leading to an increase in osmotic pressure within the endosome. This elevated osmotic pressure causes water to move from the cytoplasm into the endosome, a process that continues until it exceeds the endosome's capacity to contain water, ultimately resulting in endosomal rupture and the release of entrapped substances.

CQ does not demonstrate remarkable efficacy in suppressing the innate immune response to IVT mRNA. Drews et al. [169] transfected HFF1 cells with 4 µg of LF and 4 µg of GFP mRNA. Cells were treated with 5, 50 or 100 µM of CQ for 1 h before transfection, during RNA-cell incubation, or at 24 h post-transfection. After 24 h of transfection, cells were harvested, and RNA was isolated for global gene expression analysis based on microarrays and qRT-PCR analysis of immune response-related gene regulation. The results showed that CQ exhibited strong concentration-dependent cytotoxicity. Additionally, although the expression levels of some innate IFN response-related genes (RIG-I, OAS1, C—C Motif Chemokine Ligand 5 [CCL5], ISG20) were slightly reduced after CQ treatment, these reductions were not sufficient to counterbalance the cytotoxic effects of this compound.

Although CQ cannot inhibit the immune response induced by mRNA, it has a relatively significant enhancing effect on the translation of IVT mRNA lipid complexes in practical applications. Tusup et al. [273] used luciferase and ZsGreen as reporter genes to investigate the impact of CQ concentration and application time on mRNA expression in CT26 mouse colon cancer cells using MessengerMAX for transfection. They divided the experimental groups based on the timing of drug administration: (i) pretreatment with CQ for 1 h, (ii) simultaneous treatment with CQ and the lipid complex, and (iii) treatment with CQ after a 2-h incubation with the lipid complex, with CQ concentrations ranging from 0.15 to 5 mg/ml. The results indicated that the addition of CQ before or during transfection did not enhance mRNA expression. However, the addition of low-dose CQ (0.15–1.25 mg/ml) after a 2-h transfection period led to an increase in ZsGreen expression. Similarly, in *ex vivo* experiments, mice injected intravenously with FLuc mRNA (prepared in Mirus transfection reagent, 1–5 µg per mouse) and subsequently treated with low-dose CQ (50 µg per mouse) after 2 h showed twice the luciferase expression within 5 h post-inoculation compared to mice not receiving CQ, lasting up to 24 h. Overall, CQ has certain potential in assisting mRNA translation, with the specific performance largely depending on the dosage and timing of administration; an excessive amount of CQ does not necessarily yield better results.

##### 3.3.1.2 Acid-sensitive surfactants

Despite the fact that CQ has certain enhancement effects in mRNA translation, it usually requires multiple administrations and is limited by systemic toxicity in applications such as gene editing [274]. Therefore, there is a need for more widely applicable low molecular weight endosomal escape enhancers. Addressing this issue, Røise et al. [275] developed cage-like acid-sensitive surfactants, leveraging the membrane-disrupting ability inherent in surfactants such as Triton X. They combined reversible PEGylation with Triton X, utilizing acid-degradable acetal bonds to link short PEG chains with its hydrophobic domain, imparting pH-responsive properties, thus obtaining the first-generation cage-like surfactants capable of disrupting endosomal membranes in response to pH changes. Building upon this, to enhance affinity with nucleic acids, the research group conjugated amine moieties or RNA-binding dyes to the distal end of their PEG chains, resulting in the second and third generations of cage-like surfactants. Among these, the second generation positively charged cage-like surfactants (PCS) can further bind nucleic acids through electrostatic and hydrophobic interactions. They validated this approach through *in vitro* experiments, where in the process of transfecting human umbilical vein endothelial cells (HUVEC) with CleanCap eGFP mRNA (0.5 µg/ml, using Lipofectamine 2000 transfection reagent), the PCS negative (-) group showed minimal cell transfection, while the PCS (+) group could transfect ∼58% of cells, demonstrating a significant enhancement in mRNA translation to HUVEC by PCS. This strategy addresses several limiting factors hindering the development of current endosomal escape enhancers, such as high toxicity and low excretion, and is amenable to optimization using traditional medicinal chemistry approaches.

##### 3.3.1.3 Manganese (Mn)

A strategy for mRNA vaccine delivery involves the use of STING agonists to promote the maturation of antigen-presenting cells (APCs). Mn, an essential element in numerous physiological processes within the human body, also functions as a STING agonist [276]. It can directly activate cyclic GMP-AMP synthase (cGAS) to synthesize 2′,3′-cyclic GMP-AMP (2′,3′-cGAMP), thereby stimulating the STING pathway [277]. Fan et al. [278] constructed a highly immunogenic novel mRNA delivery system, IC8/Mn LNPs, based on a newly synthesized ionizable lipid (IC8) and the STING agonist MnCl_2_.The research team used GFP mRNA as a reporter gene to assess whether the introduction of Mn^2+^ would affect mRNA vaccine expression in DC2.4 cells and bone marrow-derived dendritic cells (BMDCs). The experiments revealed that compared to the control group (IC8@GFP), the fluorescence intensity of IC8/Mn@GFP with varying Mn^2+^ content (Mn-to-mRNA mass ratios of 0, 2.5, 5 and 10 changed to 0, 0.5, 1 and 2) increased. The transfection efficiency ranged from 20% to 30%. Additionally, Mn^2+^ application not only promoted the maturation of APCs by activating the STING pathway but also improved mRNA expression by enhancing pH buffering capacity to facilitate lysosomal escape. However, higher Mn^2+^ content did not lead to better outcomes and instead reduced mRNA transfection efficiency. This may be attributed to excessive activation of the STING pathway, resulting in excessive IFN-β production, which inhibits mRNA translation. Based on the promising results from *in vitro* experiments, the research team subsequently conducted *in vivo* experiments to investigate the distribution and expression of IC8@Luc and IC8/Mn@Luc following intramuscular injection in BALB/c mice, using Luci mRNA as a reporter gene. The results demonstrated that, compared to IC8@Luc, IC8/Mn@Luc significantly increased the bioluminescence intensity in the spleen. At the site of intramuscular injection, IC8/Mn@Luc exhibited the highest bioluminescence intensity. Overall, the incorporation of Mn enhanced the performance of mRNA translation both *in vitro* and *in vivo*, showing good safety profiles.

##### 3.3.1.4 Polyphenols

Polyphenols are a class of naturally occurring compounds characterized by the presence of multiple hydroxyl groups attached to aromatic carbons within a given molecule. This unique structural feature endows polyphenols with a high affinity for interacting with biomolecules such as proteins, polysaccharides, and nucleic acids [[279], [280], [281]]. Studies have shown that polyphenolic compounds, such as tannic acid and sialic acid, also exhibit promising performance in the field of mRNA translation, assisting in endosomal escape of mRNA and enhancing transfection efficiency.

**Tannic Acid:** Ma et al. [282] proposed the inclusion of polyphenols such as tannic acid (TA) in mRNA-LNP formulations to promote endosomal escape. The research team quantified the promotion of endosomal escape by TA(+) mRNA-LNP using two different methods through confocal microscopy. Subsequently, they conducted *in vitro* studies on FLuc mRNA expression and cell viability in Madin-Darby Canine Kidney (MDCK) cells (as some RNA drugs accumulate in the kidneys in the absence of carriers). The experimental results demonstrated that the addition of TA to the formulation improved the endosomal escape of mRNA-LNP and ultimately enhanced mRNA expression.

**Sialic acid:** Dendritic cells (DCs) are the only APCs capable of activating naive T cells, with just one DC capable of activating 100–200 T cells, making them a critical target for vaccine development [[283], [284], [285]]. Tang et al. [286] developed a sialic acid (SA) cholesterol derivative modified mRNA vaccine SA-Pchs-LNPs (Pchs is MPEG_2000_-hemicsuccinate cholesterol that can be cut by carboxylesterases *in vivo*). This modification allows the nanocarriers to evade the issue of reduced efficacy upon repeated intravenous injection and further enhances the targeting efficiency of DCs. Specifically, through the interaction between SA and SA-binding immunoglobulin-like lectin-1 (siglec-1), widely expressed on DCs, this approach enables rapid capture of the mRNA vaccine by DCs. Moreover, delivery of mRNA can upregulate siglec-1 on DCs, thereby augmenting the vaccine's ability to specifically target DCs at the site of injection. They initially conducted *in vitro* experiments, incubating DC2.4 cells or BMDCs with FLuc mRNA-LNP for 24 h, and measured Fluc levels using a Fluc detection kit. The results indicated that SA-Pchs-LNPs demonstrated the highest transfection efficiency, with relative luminescence units (RLU) 2–3 times higher than that of 1.5Pdmg-LNP, a commercial LNP formulation. Co-localization analysis of DiD-labeled LNPs and lysosomes revealed the lowest overlap signal for SA-Pchs-LNPs and lysosomes (<10%), whereas other groups (*e.g.*, 1.5Pdmg-LNPs) showed a 40%−60% co-localization rate at 120 min, highlighting the excellent endosomal escape capability of the SA-modified mRNA vaccine. Further *in vivo* experiments assessed the therapeutic effect of SA-modified mRNA vaccine on B16-OVA tumor-bearing mice via intravenous injection. The results showed that even at a low dose, SA-Pchs-LNPs/ovalbumin (OVA) mRNA could inhibit 70% of B16-OVA tumors, significantly outperforming 1.5Pdmg-LNPs/OVA mRNA.In summary, the design of SA-modified mRNA vaccine simultaneously achieved DC targeting and effective endo/lysosomal escape. Over 90% of SA-modified LNPs could rapidly escape from early endosomes (EEs) to avoid entry into lysosomes, thereby allowing translation of mRNA in the cytoplasm and endoplasmic reticulum, doubling the expression of target proteins in DCs, and ultimately producing excellent antitumor therapeutic effects with minimal side effects.

##### 3.3.1.5 Oligopeptide: GALA

mRNA-based vaccines have become effective systems for eliciting T-cell responses against a variety of cancers and infectious diseases caused by viruses. However, the selective and efficient delivery of mRNA to APCs remains a major challenge in the development of mRNA vaccines. To address this issue, Lou et al. [287] screened three different peptides, namely LEDE [sequence: (2-azido)-IGKEFKRIVERIKRFLRELVRPLR-OH], Melittin [sequence: (2-azido)-GIGAVLKVLTTGLPALISWIKRKRQQ-OH], and GALA [sequence: (2-azido)-WEAALAEALAEHLAEALAEALEALAA-OH]. Experimental results showed that compared with the commercial lipid transfection reagent Lipofectamine 2000, the polyplexes modified with GALA (PPx-GALA) could efficiently deliver EGFP mRNA to macrophages and DCs with higher transfection levels. Specifically, DC2.4 cells exhibited moderate EGFP expression (∼20%) when transfected with Lipofectamine 2000, while the transfection efficiency increased to 28% with the PPx-GALA formulation, without inducing significant cytotoxicity. Furthermore, researchers investigated the cellular uptake mechanism and intracellular trafficking of PPx-GALA and found that GALA peptides have a dual function: they can selectively bind to polysaccharides at the SA termini on DCs and promote the disruption of endosome/lysosomal membranes, leading to internalization and subsequent cytosolic release. Overall, this is the first study to apply GALA-modified polyplexes to facilitate mRNA delivery to APCs, and PPx-GALA can promote endosomal escape and enhance transfection efficiency.

##### 3.3.1.6 Charge-conversion polymer

Dirisala et al. [288] approached the problem from a novel perspective by integrating the fine-tuning of conjugate chemistry with endosomal escape mechanisms, integrating two complementary functions into a single polymer, specifically poly(L-ornithine) (PLO)/mRNA polyplexes encapsulated by an innovative charge conversion polymer (CCP). This polymer bears a negative charge at extracellular pH values (7.3) but converts to a positive charge at the acidic pH values of endosomes (5.5) to disrupt the endosomal membrane. They utilized confocal laser scanning microscopy (CLSM) to assess the endosomal escape capability of this formulation, confirming the effectiveness of CCP-based thermosensitive sulfonamide elastomers in escaping from endosomes. Subsequently, the mRNA expression efficiency of polyplexes was measured in HUVECs using mRNA encoding Gaussia luciferase (GLuc). The experimental results indicated that compared to naked mRNA, the PLO group exhibited a 38-fold increase in GLuc expression after 24 h of mRNA treatment. In summary, this design significantly enhances the transfection efficiency of mRNA by promoting endosomal escape.

#### 3.3.2 Endosomal recycling inhibitors

In addition to promoting endosomal escape, inhibiting endosomal recycling can also enhance the efficiency of mRNA translation. To this end, Shin et al. [289] screened a library of small molecules targeting intracellular transport to characterize LNP-mediated mRNA delivery. They ultimately identified two effective small molecules, NAV2729 (NAV) and endosidine 5 (ES5), which significantly enhance mRNA expression by inhibiting intracellular endocytic recycling. Specifically, a quantitative assessment of the impact on Fluc expression after delivering Fluc mRNA LNPs to HEK293T cells for 24 h showed that the small molecules NAV and ES5 enhanced mRNA expression by 1.5-fold and 2-fold, respectively, with further enhancement observed when NAV and ES5 were co-incubated. They explored the mechanism of enhancement using a membrane remodeling sensor and found that both NAV and ES5 target the endocytic recycling pathway, with ES5 being an effective and specific inhibitor of the membrane-associated protein annexin A6 (ANXA6), and NAV being an effective inhibitor of ADP-ribosylation factor 6 (ARF6)-dependent endocytic recycling. Building on this, they conducted *in vivo* experiments, incubating NAV or ES5 with Fluc mRNA-LNP and administering it via intramuscular injection in BALB/c mice, and quantified Fluc expression using bioluminescence imaging. The results showed that NAV significantly improved the translation efficiency of Fluc mRNA-LNP, while ES5 did not exhibit enhancement. Overall, the strategy of inhibiting endosomal recycling can also reduce mRNA degradation and enhance mRNA transfection.

### 3.4 RNase inhibitors

In addition to the aforementioned endosomal degradation, mRNA within cells is also subject to degradation by endogenous RNases. The OAS/RNase L system, introduced in the previous section, is an innate immune pathway that responds to dsRNA in the form of PAMPs, inducing the degradation of viral and cellular RNA, thereby blocking viral infection [290]. Specifically, upon activation by dsRNA-PAMP, certain OAS proteins generate 2–5A from cellular ATP. 2–5A is a unique ligand that can bind with high affinity to monomeric and latent RNase L, leading to the dimerization and activation of RNase L [291]. Active RNase L can cleave various ssRNA substrates, including viral genomes and cellular RNA, directly impacting protein synthesis and restricting viral replication [292]. Therefore, to mitigate the impact of RNases during mRNA translation, the use of corresponding RNase inhibitors is under consideration.

#### 3.4.1 Protein inhibitors: RNasin

The protein inhibitor of RNase (RNasin) is an acidic ribonucleoprotein extracted from rat liver or human placenta. It is a non-competitive inhibitor of RNase, capable of binding to RNase through non-covalent bonds, rendering it inactive [293,294]. RNasin is non-toxic and widely used as an effective RNase inhibitor [295,296]. Of course, as a protein, it is not a small molecule but a large one. For the sake of logical coherence in this article, it is categorized as such.To address the issue of low reproducibility when locally administering naked sa-RNAs in the skin or muscle using electroporation, Huysmans et al. [297] combined sa-RNAs with protein-based RNase inhibitors. Experimental data showed that supplementing naked sa-RNAs with RNase inhibitors before intradermal electroporation increased median total luciferase expression by 70-fold and improved the success rate of the administration procedure from 33% to 100%. These results were reproducible across experiments. Furthermore, owing to the proteinaceous nature of RNasin, it imposes relatively stringent requirements in terms of pH and handling. At pH levels below 6, RNasin starts to lose its ability to inhibit RNAnse, as its inhibitory action relies on electrostatic interactions between the negatively charged RNase inhibitor (at physiological pH) and the positively charged RNase (at physiological pH). The inhibitory effect of RNasin reaches its maximum between pH 7 and 8. In terms of handling, to protect naked mRNA from endogenous RNase degradation, RNasin should be added before administration and should not be stored together with mRNA in the refrigerator [297].

#### 3.4.2 Other potentially effective RNase inhibitors

Although RNasin has shown good performance in the field of mRNA translation, its stringent usage conditions have somewhat limited its expanded application. In addition to RNasin, there are numerous RNase inhibitors that, as small molecule drugs, may offer more convenient usage conditions. However, there is currently a lack of exploration regarding their application in the mRNA field, representing a potentially fruitful area for further investigation.

**Sunitinib**: Sunitinib is a tyrosine kinase (TYK) inhibitor of vascular endothelial growth factor receptors (VEGFR) and platelet-derived growth factor receptors (PDGF-R), currently clinically used to suppress renal cancer by blocking angiogenesis [298,299]. In addition, Jha et al. [300] reported the *in vivo* inhibition of PKR and RNase L by sunitinib with IC_50_ values of 1.4 and 0.3 µM, respectively. Molecular dynamics simulations and dynamic light scattering results by Tang et al. [301] suggest that binding of sunitinib at the PKR structural domain destabilizes the dimerization conformation of RNase L. Based on this, Jha et al. [302] explored the potential of combining sunitinib with oncolytic viruses to enhance viral replication in tumor tissues. They observed that the sunitinib/oncolytic virus combination inhibited eIF2α phosphorylation, increased viral replication in tumors, and resulted in tumor regression. These results suggest that the transient inhibition of innate immunity by sunitinib can enhance oncolytic virotherapy, leading to the recovery of tumor-bearing animals. This also demonstrates the potential application of sunitinib in improving expression in the mRNA field.

**Thienone compounds:** Hwang et al. [303] employed structure-based rational design to develop novel RNase L inhibitors. By conjugating 1H-pyrrole-3-carboxamide with 2-aminothiophen-4-one, they obtained the scaffold 2-((pyrrol-2-yl)methyl)thiophen-4-one and synthesized 34 derivatives. All compounds were initially subjected to *in vitro* fluorescent resonance energy transfer (FRET) assays at concentrations of 250 or 130 µM to assess their potency as RNase L inhibitors. The results indicated that compounds 17a (JH259), 17c (JH332), and 17d (JH333) exhibited a 30-fold increase in inhibitory efficacy compared to sunitinib, possibly due to their extensive molecular interactions with key residues around the ATP-binding pocket and the three-dimensional complementarity between thienone compounds and the ATP-binding pocket. These thiazole compounds represent some of the most potent synthetic small molecule RNase L inhibitors reported to date, providing a series of thiazole-based chemical tools for modulating RNase L activity, and are potential compounds for reducing the degradation of delivered mRNA.

**Curcumin:** Rath et al. [304] discovered that curcumin, a natural anti-inflammatory compound, is a specific and potent inhibitor of human RNase L. It can non-competitively inhibit RNase L, possibly by inducing conformational changes in the enzyme, resulting in complete loss of its activity.

**Ellagic acid and valerianic acid lactone:** Daou et al. [305] conducted an *in vitro* screening of a library containing 500 protein kinase inhibitors to identify drugs that regulate RNase L activity. They discovered that ellagic acid exhibited a tenfold higher selectivity towards RNase L compared to its closest homolog, immunoglobulin-regulated enhancer 1 (IRE1). Furthermore, they identified valerianic acid lactone as a superior inhibitor of RNase L, exhibiting a 100-fold higher selectivity compared to IRE1.

**Myricetin:** Tang et al. [306] identified 13 inhibitory fragments targeting RNase L through enzymatic assays and NRM screening. Among these, three natural products—myricetin, quercetin, and kaempferol—share a similar scaffold with fragment AC40357 and exhibited effective inhibitory activity *in vitro*, with myricetin demonstrating notable cellular inhibitory activity. The co-crystal structure of RNase L with myricetin provides a structural basis for inhibitor design by modulating ribonuclease activity through conformational regulation.

## 4 Discussion

This review systematically organizes the latest progress in the codelivery of mRNAs and translation booster molecules. These molecules can be classified into two categories, macromolecules and small molecules, according to their molecular size. The aim of administering translations boosters is to reduce associated side effects and improve translation efficiency through strategies such as inhibiting the immune response triggered by the mRNA and promoting endosomal escape (Table 3).

### 4.1 Future prospects

**Fig. 4:**
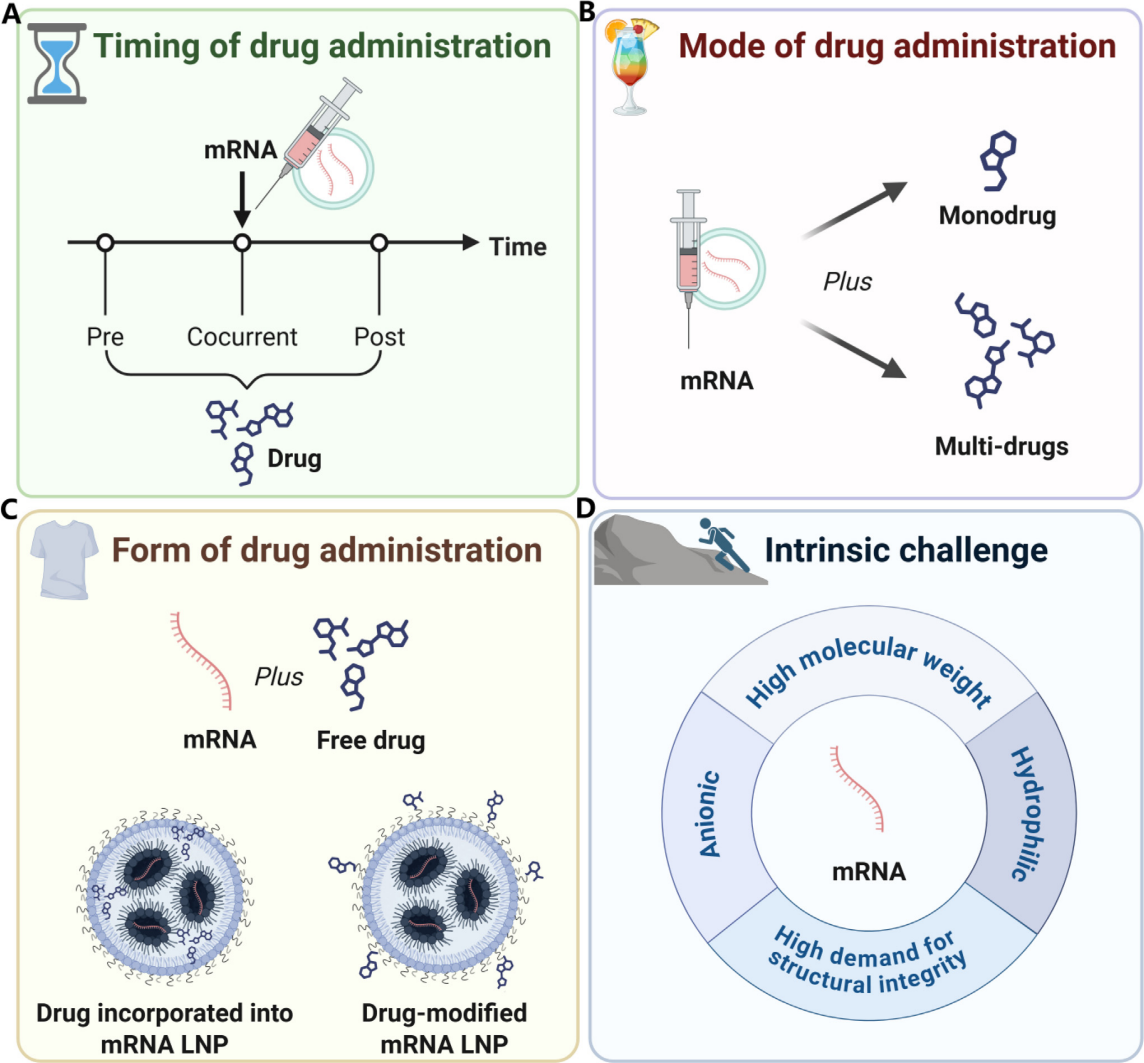
Four factors are involved in modulating the immunogenicity and translational efficacy of therapeutic mRNAs and pharmaceutical agents. **(A**) Timing of drug administration: Concurrent injection, pretreatment, or posttreatment with drugs. **(B**) Mode of drug administration: Compared with single-drug treatment, the combined application of multiple regulatory drugs may further enhance the efficacy of mRNA therapy. **(C**) Form of drug administration: Incorporating free drugs directly into the carrier or conjugating them to the carrier may serve as a more efficient approach. **(D**) Inherent challenges in mRNA delivery: The success of mRNA therapy lies in ensuring the structural integrity and efficient delivery of large, anionic and hydrophilic mRNA to the target. This graphic was created with BioRender.com.

Upon comprehensive analysis, we conclude that the efficacy of these mRNA translation-enhancing molecules depends on the dosage, timing, and route of administration, and that the combined application of multiple strategies can, in some cases, yield synergistic effects (Table 1, Table 2, specifically Fig. 4).

**Table 1:** Enhanced expression strategies – macromolecules.

Drug class	Drug name	Mechanisms	Experimental program		Specific improvement in effectiveness			Ref
			*In vitro* experiment	*In vivo* experiment	Immune suppression	Translation enhancement	Safety	
Natural viral proteins	VACV: B18R protein	An IFN decoy receptor neutralizes type I IFNs and inhibits IFNAR activation and subsequent pathway signaling	HFFswere transfected with mRNA encoding TF (*e.g.*, OCT4, SOX2, KLF4) using RNAiMAX; B18R was added before, during, and after delivery of the mRNA complexes, respectively; the results of the relevant gene examination were detected by qRT-PCR	/	B18R inhibited the innate immune response activated after non-viral mRNA transfection but did not induce down-regulation of immune response-related genes	/	/	[169]
			HFFs were transfected with a reprogrammed mRNA mixture using a lipid transfection technique	/	B18R inhibited >90% of OAS1 induction owing to its neutralization of the IFN	/	/	[168]
			HUF1s were transfected with OCT4 mRNA using RNAiMAX and B18R recombinant protein was added to the culture medium	/	/	There was no statistically significant increase in OCT4 expression, and no significant increase was observed after further use of higher amounts of synRNA, B18R or both	/	[170]
	VACV: E3 protein	Efficient blocker of PKR activation and IFN-β upregulation	HFFs were co-transfected with E3 mRNA and GFP sa-RNAs by electroporation or liposomal transfection techniques	/	E3 prevented the phosphorylation of PKR and eIF2α and significantly reduced about 90% of IFN-β-induced	/	No improvement in cell viability by E3 was observed	[172]
			HFFs were co-transfected with GFP sa-RNAs, iRFP mRNA and E3 mRNA; sa-RNAs encoding secretable deep-sea shrimp luciferase was used for further validation	/	/	Co-transfection of E3 mRNA resulted in a significant 3- to 35-fold increase in the expression of target gene	/	[172]
			ASCs were co-transfected with SEC combination using DIM1T LNP	Delivery of HGF and CXCL12 in a diabetic skin wound model using the DIM1T LNP-SEC engineering ASCs	SEC significantly reduced the expression levels of phosphorylated PKR and eIF2α in ASCs	SEC enhanced luciferase expression and this expression could be maintained for at least 48 h after treatment, and the use of DIM1T LNP-SEC engineered ASCs, which prolonged the expression of HGF and CXCL12 in a diabetic skin wound model, showed superior wound healing efficacy		[175]
	VACV: EKB protein	Co-application of immune evasion proteins	HFFs were co-transfected with EGFP mRNA and EKB mRNA by lipid transfection technique	/	When E3 and K3 mRNA were co-transfected, IFN-β transcript levels were reduced by 80%, and IFN-β transcript levels were further reduced after addition of B18R mRNA or rB18R	When co-transfected with E3 and K3 mRNA, this resulted in a three-fold increase in translation of EGFP mRNA, which was increased to 4.6-fold upon additional treatment with B18R mRNA or rB18R. Further, repeating the experiments with Luci mRNA, EKB mRNA again significantly increased translation, highlighted by a substantial prolongation of translation time, and higher Luciferase expression in EKB samples compared to controls 72 h after transfection	Unprotected HFFs transfected 4 times 1 d with non-modified mRNA died, whereas co-transfection with EKB mRNA rescued the survival of HFFs	[168]
			EKB mRNA and GFP sa-RNAs were co-transfected into HFFs with RNAiMAX or MessengerMax	Using immunocompetent BALB/c mice, the RNA was injected intramuscularly into the thighs, either FLuc sa-RNAs alone, or in combination with EKB mRNA	Co-transfection of EKB mRNA reduced IFN-β induction by 95%	In control mice, the signal was barely detectable by *in vivo* bioluminescence imaging, but in mice co-injected with EKB mRNA, high-intensity luminescent signals were detected at the injection site for more than 2 weeks	/	[172]
	IAV: NS1 protein	Interferes with innate immunity and evades host defense monitoring	BJ fibroblasts were co-transfected with NS1-TX91 mRNA and luciferase mRNA or GFP mRNA (control) using Stemfect	/	Cells transfected with NS1 mRNA produced less IFN compared to GFP mRNA control	Co-delivery of mRNA encoding NS1-TX91 mediated higher transfection enhancement	NS1-TX91 had a negative effect on cell viability, but it can be considered tolerable at normal transfection doses (>80% viability)	[181]
			/	Low-dose nude luciferase/NS1-TX91 mRNA (2 µg/2 µg) was injected subcutaneously into the auricle and tail base of ICR mice	/	The initial co-administration did not elevate the expression of luciferase to a statistically significant level. However, when a second dose was co-administered at the same site 20 h later, the mice that received the co-injection exhibited higher luciferase expression compared to the control group (luciferase/GFP mRNA)	/	[181]
			BJ fibroblasts, HepG2, RAW 264.7, pMEF and Vero cells were co-transfected with NS1 mRNA and GFP mRNA or luciferase mRNA (control) using Stemfect	/	/	Co-transfection of NS1 and GFP mRNA rapidly eGFP expression in every cell type capable of IFN production, with no observed enhancement in IFN-deficient Vero cells	A slight increase in survival was observed for NS1-HK97 in all cell groups, suggesting that it could attenuate the toxicity induced by mRNA transfection	[182]
			C3H10T1/2 were co-transfected with NS1 mRNA and BMP-2 mRNA using MessengerMax	/	/	When the mass ratio of BMP-2 mRNA to NS1 mRNA was 3:1, the expression of BMP-2 was the highest and increased 8-fold	/	[184]
	MERS-CoV ORF4a protein	Interference with innate antiviral signaling pathways, including NF-κB-mediated responses	Using pABOL as a delivery vector, the Fluc sa-RNAs of the MERS-CoV ORF4a construct was delivered into both HeLa and MRC5 cell lines	Validated in C57BL/6 mice (high producers of IFN-α/β and IFN-γ)	/	*In vitro* validation increased FLuc expression by 893-fold and 109-fold, respectively; *in vivo* validation showed a slight increase in the protein expression of FLuc	/	[188]
	PIV-5 V protein	Antagonizing the host's antiviral response	Using pABOL as a delivery vector, the Fluc sa-RNAs of the PIV-5 V construct was delivered into both HeLa and MRC5 cell lines	/	/	The expression of sa-RNAs increased by 796-fold and 72-fold, respectively	/	[188]
			/	A clinically relevant human skin explant model was selected for *in vivo* validation, with EGFP sa-RNAs as the reporter gene	/	The percentage of EGFP+ cells increased linearly with increasing sa-RNAs dose, and PIV-5 V enhanced expression in immune cells by increasing the percentage of cells expressing sa-RNAs	/	[188]

**Table 2:** Enhanced expression strategies - small molecules, short peptides and proteins.

Drug class	Drug name	Mechanisms of action	Experimental program		Specific improvement in effectiveness			Ref
			*In vitro* experiment	*In vivo* experiment	Immune suppression	Translation enhancement	Safety	
Anti-inflammatory drugs	DexDex	Glucocorticoid receptor agonist	LNPssPalm was used as a delivery vehicle to deliver Fluc mRNA to pMEFs, and Dex was also added	/	/	Dex caused an increase in transfection activity mainly in the middle and late stages of transfection (4–48 h), ultimately resulting in a 1.6-fold increase in transfection activity	Dex readily dissociated from LNP in the circulation, leading to the risk of systemic exposure of steroid drugs	[223]
	Dex-Pal	Achieving *in vivo* co-delivery of IVT-mRNA and Dex to the same cells/organs	/	Dex-Pal was formulated into Luci mRNA-LNP and injected intravenously into BALB/c mice and C57BL6/J mice	/	Dex-Pal successfully increased protein expression in mouse liver by 2.7-fold in BALB/c mice and 6.6-fold in C57BL6/J mice	/	[223]
	LD003	Enhancing the hydrophobicity of Dex reduced its dissociation from the LNP, facilitating co-delivery	/	FLuc mRNA was formulated into LNP containing 0 or 10 mol% LD003 and injected subcutaneously into C57Bl/6 mice at the interscapular space, and blood was collected and plasma cytokine levels were measured 2 h post-injection	Cytokine production in LD003-containing mRNA LNP-treated animals was greatly reduced, almost close to background levels, and had a greater immunosuppressive effect than the 20 mg/kg free Dex	/	Avoiding adverse side effects associated with high dose steroids	[224]
	LNP formulation for partial cholesterol replacement with Dex	LNP with anti-inflammatory properties	HepG2 cells were transfected with Fluc mRNA-LNP, in which a portion of cholesterol was replaced by Dex	/	/	There was no reduction in transfection efficiency of C9D1 LNP compared to C10D0 LNP	Did not show increased cytotoxicity	[226]
			RAW246.7 cells were treated with C10D0 LNP and C9D1 LNP for 24 h to assess their anti-inflammatory effects	/	C9D1 LNP treatment halved the increased levels of TNF-α	/	/	[226]
			/	Using C57BL/6 mice to investigate the inflammatory response and mRNA delivery efficacy of the anti-inflammatory LNP formulation	Compared with C10D0 LNP-treated mice, C9D1 LNP-treated mice had significantly lower serum TNF-α concentrations and successfully reduced LNP-induced inflammatory responses *in vivo*	The transfection efficiency was significantly enhanced by 1.5-fold	/	[226]
	Proester of roflupenil and budesonide	Glucocorticoid receptor agonist	/	Using a LNP formulation incorporating prodrugs of roflumilast and budesonide, hFGF21 mRNA was delivered via intravenous injection into CD1 mice	/	Continued increase in protein production levels; higher overall whole-body protein exposure	Reduced risk of high-dose exposure to steroid drugs	[225]
	Clobetasol and hydrocortisone	Topical administration of glucocorticoid receptor agonist	/	Topical application of clobetasol and hydrocortisone ointment at the injection site inhibited the type I IFN response induced by ZIKVac-sa-RNAs vaccine	Clobetasol and hydrocortisone were effective in inhibiting the type I IFN response, and in combination with BAY11 can further reduce the level of response	Hydrocortisone improved translation *in vivo* on Day 2 and 3 post-injection, with clobetasol performing better, prolonging translation by 1 week and increasing overall translation of mRNA by 3.5-fold	Clobetasol prevented the formation of antibodies against sa-RNAs-encoded antigens and should therefore be avoided in the vaccination setting	[229]
Promoting endosomal escape	CQ (antimalarial drug)	Proton sponge effect; Electrostatic interactions; Osmotic pressure	GFP mRNA was complexed with RNAiMAX for transfection; HFF1 cells were incubated with 5, 50, and 100 µM CQ 1 h prior, during, or 24 h post-transfection; the cells were harvested to isolate the RNA for microarray-based global gene expression analysis and qRT-PCR to analyze the immune response-associated gene regulation	/	Expression levels of some innate IFN response-associated genes (RIG-I, OAS1, CCL5, ISG20) were slightly reduced, but these reductions were not sufficient to offset the cytotoxic effects of the molecule	/	CQ had a strong concentration-dependent cytotoxic effect	[169]
			Fluc mRNA and ZsGreen mRNA were used as reporter genes; CT26 cells were transfected using MessengerMAX; CQ was added to test the effect of drug concentration and application time on IVT mRNA expression	/	/	Supplementation of CQ before or at the time of transfection did not enhance IVT mRNA expression, and a low dose (0.15–1.25 mg/ml) of CQ added 2 h after transfection resulted in increased expression of ZsGreen	/	[273]
			/	Mice were intravenously injected with FLuc mRNA and received a low dose (50 µg per mouse) of CQ 2 h later	/	Expression of luciferase was twice that of mice that did not receive CQ within 5 h after vaccination and was observed for 24 h	/	[273]
	Acid-sensitive surfactants	Acid-sensitive surfactants have membrane-disrupting ability, disrupting endosomal membranes in response to pH changes and further binding nucleic acids through electrostatic and hydrophobic forces	PCS (2 µg/ml) was added to CleanCap eGFP mRNA (0.5 µg/ml), and HUVECs were transfected using Lipofectamine 2000 after incubation for 30 min at room temperature	/	/	Almost no cells were successfully transfected in the PCS^-^ group, whereas the PCS^+^ group was able to transfect ∼58% of the cells, suggesting that PCS significantly enhances the delivery of mRNA to HUVECs	Not highly toxic	[275]
	Mn	Mn is an IFN agonist that stimulates the STING pathway	Mn^2+^ was introduced into ionizable lipid IC8 LNPs with GFP mRNA used as a reporter gene to assess whether Mn affects mRNA vaccine expression in DC2.4 cells and BMDCs	/	/	The fluorescence intensity of IC8/Mn@GFP with different manganese content was increased with transfection efficiencies of 20% to 30% compared to the control (IC8@GFP) which was not successfully transfected. The optimal Mn content for mRNA transfection efficiency is not simply the highest, as excessively high levels can actually hinder transfection by triggering over-activation of the STING pathway. This excessive activation leads to an upregulation of IFN-β production, which in turn inhibits mRNA translation.	/	[278]
			/	The distribution and expression of IC8@Luc and IC8/Mn@Luc were studied in BALB/c mice under intramuscular injection	/	Compared with IC8@Luc, IC8/Mn@Luc significantly increased the bioluminescence intensity in spleen, and the highest bioluminescence intensity was observed at the intramuscular injection site, suggesting that IC8/Mn LNPs performed well in delivering mRNA *in vivo*	Good safety	[278]
	TA	TA are polyphenols that incorporate multiple hydroxyl groups, rendering them a high affinity for interacting with biomolecules such as proteins, polysaccharides and nucleic acids	TA was incorporated into mRNA LNPs, and studies on Fluc mRNA expression and cell viability are conducted *in vitro* using MDCK	/	/	Incorporation of TA into the formulation improved endosomal escape of mRNA-LNP and ultimately promoted mRNA expression	/	[282]
	SA	SA-Pchs-LNPs were rapidly captured by DCs through the interaction of salivary acid with siglec1, which also up-regulated siglec1 on DCs to enhance the DCs-targeting ability of the vaccine at the injection site	Fluc mRNA was encapsulated with SA-Pchs-LNPs and incubated with DC2.4 cells or BMDCs for 24 h. The Fluc level of the cells was detected by Fluc detection kit	/	/	SA-Pchs-LNPs exhibited the highest transfection efficiency in both cell lines, 2–3 times higher than commercial LNPs preparations; >90% of SA-modified LNPs could rapidly escape from EEs, and mRNAs were translated simultaneously in the cytoplasm and ribosomes of the endoplasmic reticulum, which doubled the expression of target proteins in DCs.	/	[286]
			/	Therapeutic efficacy of SA-modified mRNA vaccine injected intravenously in B16-OVA tumor-bearing mice	/	SA-Pchs -LNPs/OVA mRNA inhibited 70% of B16-OVA tumors at a low-dose regimen, which was significantly better than 1.5 Pdmg-LNPs/OVA mRNA	SA-modified vaccines were more immunogenic	[286]
	GALA peptide	GALA peptide selectively binds to SA-terminated glycans on DCs, leading to internalization and subsequent cytoplasmic release by promoting endo/phagosomal membrane disruption	Using PPx-GALA as a delivery vector and Lipo as a control vector, EGFP mRNA was transfected into macrophages and DCs	/	/	When transfected with Lipo, ∼20% EGFP expression level was observed in DC2.4 cells, and a transfection efficiency of 28% could be achieved with the PPx-GALA preparation, suggesting that PPx-GALA has a higher transfection level	No significant cytotoxicity	[287]
	CCP	CCP is negatively charged at extracellular pH 7.3 but shifts to a positive charge at endosomal acidic pH 5.5 to disrupt endosomal membranes	Using CCP as the delivery vehicle, the transfection efficiency of Gluc mRNA in HUVECs was measured	/	/	Compared to naked mRNA, the PLO group resulted in a 38-fold increase in the expression of GLuc after 24 h of mRNA treatment	/	[288]
Inhibition of endocytic recycling	NAV2729 and Endosidine 5	ES5 is a potent inhibitor of the specific membrane-bound protein ANXA6 lipid interaction; NAV is a potent inhibitor of the ARF6-dependent endocytosis cycle	Encapsulated within the current COVID-19 vaccine LNP with Fluc mRNA, the transfection of HEK293T cells was performed, followed by a 24 h incubation with NAV and ES5 to assess the impact of these small molecules on Fluc expression	/	/	NAV and ES5 enhanced mRNA expression by 1.5- and 2-fold, respectively; co-incubation with NAV and ES5 further increased expression	/	[289]
			/	NAV or ES5 was incubated with Fluc mRNA-LNP and injected intramuscularly in BALB/c mice; Fluc expression was quantified using bioluminescence imaging	/	NAV significantly enhanced the delivery efficiency of Fluc mRNA-LNP, whereas, in contrast, ES5 did not have an enhancing effect, probably because its inherent carrier activity impeding mRNA-LNP delivery *in vivo*	/	[289]
Inhibition of inflammatory signaling pathway	ODN20958, ODN2088	ODN20958 and ODN2088 disrupt co-localization of CpG ODNs with TLR9 in endosomes, thereby inhibiting IFN-α and IL-6 release	/	In IFN-β luciferase reporter mice, ODN2088 and ODN20958 were co-administered with the ZIKVac-sa-mRNA vaccine	Co-injection of these two TLR inhibitors with the ZIKVac-sa-RNAs vaccine significantly reduced immediate type I IFN responses; however, this immunomodulatory effect disappeared after 1 d and exhibited a more restricted impact when administered immediately following mRNA administration	/	/	[229]
			/	In BALB/c mice, Luci sa-RNAs was delivered via intradermal electroporation, with the combined application of ODN2088 and ODN20958	/	*In vivo* translation was not improved when ODN2088 was applied alone; a better therapeutic effect was observed when topical clobetasol was combined with ODN2088 and/or BAY11	/	[229]
	BAY11	BAY11 can enhance mRNA translation efficiency by inhibiting NF-κB	OCT4 mRNA was complexed with RNAiMAX, BAY11 was added to the medium, HUF1s were transfected, and OCT4 was quantified by immunocytochemistry analysis	/	/	BAY11 resulted in robust OCT4 stability and more uniform expression at 0.01 µM and produced the highest OCT4 expression at 1 µM	1 µM of BAY11 had a significant inhibition effect on cell proliferation; 0.01 µM of BAY11 achieved the best compromise between robust OCT4 expression and no statistically significant reduction in cell proliferation	[170]
			/	In BALB/c mice, the ability of BAY11 to inhibit the *in vivo* type I IFN response induced by ZIKVac-sa-RNAs was assessed	ZIKV-sa-RNAs co-injected with high-dose (25 µg) of BAY11significantly inhibited IFN-β responses within 24 h, whereas low-dose (12.5 µg) of BAY11 was ineffective; BAY11 demonstrated significant reduction of innate immune responses only when administered directly after mRNA administration	/	/	[229]
	BX795	BX795 is a potent inhibitor of TBK1 and IKKɛ and has been shown to inhibit the phosphorylation of IRF3, which may ultimately inhibit many degradation-related cytokines, RNase L and chemokines	OCT4 mRNA was complexed with RNAiMAX, BX795 was added to the culture medium, HUF1s were transfected, and OCT4 was quantified by immunocytochemical analysis	/	/	When combined with 120 ng/µl of total synRNA, low concentrations (0.001 µM) of BX795 induced broadly stable and homogeneous expression of OCT4	BX795 exhibited pronounced impairments in cell proliferation and raised certain safety concerns	[170]
	ISRIB	ISRIB reverses eIF2 phosphorylation and restores translation by targeting eIF2B	Fluc mRNA was delivered to mouse pMEFs using LNPssPalm as the delivery vehicle, and ISRIB was added at the same time	/	/	ISRIB enhanced transfection activity mainly at the early stage of transfection (0–6 h), and early cumulative luciferase activity indicated that ISRIB enhanced transfection activity by 1.4-fold	/	[223]
	Ruxolitinib	Ruxolitinib interferes with the JAK/STAT signaling pathway, which inhibits the production of IFN effectors, suppresses the up-regulation of PRR and TFs, and prevents IFN induced effects	/	BABL/c mice were given an intravenous injection of FLuc sa-RNAs with or without MERS-CoV ORF4a, with or without co-formulation with ruxolitinib, and protein expression was quantified at 4-, 7-, 10- and 14-d post-injection	/	Ruxolitinib resulted in a modest increase in sa-RNAs protein expression	/	[188]
	TSA	TSA inhibits IRF-7 nuclear translocation and IFN-I transcription	HFF1s were transfected with GFP mRNA complexed with LF and incubated with TSA	/	No significant down-regulation of assessed immune response-related genes was observed	/	/	[169]
	Most cardiac glycosides, natural compounds that inhibit NF-κB, TLR3 inhibitors, PKR inhibitors	Small molecule inhibitors of IFN	BJ fibroblasts were pretreated with small molecule inhibitors of IFN 1 h prior to transfection, GFP mRNA was complexed with Stemfect, and transfection was incubated for 4 h, followed by detection of transfection efficiency	/	PKR inhibitors effectively inhibited IFN production	None of the small molecules tested enhanced mRNA transfection in BJ fibroblasts, and one-third of the compounds tested unexpectedly inhibited GFP expression	/	[253]
Inhibition of RNases	RNasin	RNasin is a non-competitive inhibitor of RNAase that binds to RNase in a non-covalent manner and inactivates it without toxicity, making it a very effective and frequently used RNase inhibitor	/	The study locally administered naked sa-RNAs via electroporation in the skin or muscle, concurrently using RNasin	/	Supplementation of naked sa-RNAs with RNase inhibitors prior to intradermal electroporation resulted in a 70-fold increase in median total luciferase expression and also increased the success rate of the drug delivery procedure from 33% to 100%	/	[297]

**Table 3:** Comparison of the mechanisms, limitations, effectiveness and usage scenarios of mRNA translation boosters.

Molecular weight classification	Mechanism-based classification	Mechanism of mRNA translation boosting	Limitation	Safety	Relative effectiveness of boosting translation	Application scenarios
Macromolecular drugs	Natural viral proteins	Co-deliver the mRNA together with these viral proteins that possess immune evasion properties, utilize the immune evasion ability of the virus, downregulate the immunogenic response, and enhance the regulation of mRNA translation.	Long-term use of viral proteins may inhibit antiviral immunity and increase the risk of secondary infection.	Risk of off-target immune regulation. As foreign antigens, viral proteins may be recognized by the host immune system; Risks associated with genomic integration.	EKB mRNA: Co-transfect HFF cells with unmodified synthetic mRNAs encoding EKB and eGFP. The co-transfection of EKB mRNA increases the translation efficiency of GFP mRNA to 4.6 times.	In some studies, B18R protein is used as an mRNA translation adjuvant. This is achieved by adding the rB18R as a soluble protein to the cell culture medium, or by co-transfecting it with the mRNA encoding the target protein; Efficient generation of induced iPSCs is achieved by co-transfecting the combination of EKB mRNA and miRNA.
	Short artificial RNA molecules	Selectively inactivate the corresponding mRNA of the target gene through dsRNA thereby effectively silencing or suppressing the expression of the target gene. For example, the knockout of genes such as PKR and STAT can rescue cells that trigger an innate immune response due to frequent transfection, and improve the translation efficiency.	siRNAs and shRNAs may trigger the innate immune response by inducing IFN signaling through TLR3, which, to some extent, weakens the effect of translation enhancement.	siRNAs and shRNAs may trigger innate immunity by inducing type I IFN signaling through TLR3; miRNAs have the ability to regulate the inflammatory response; circ-RNAs have higher stability and lower immunogenicity compared to linear RNAs.	There is no specific data regarding the boosting of mRNA translation.	Efficiently generate iPSCs by co-transfecting the combination of Short artificial RNA molecules and mRNA; Modify circ-RNAs into novel RNA aptamers and protein translation vectors.
Non-viral small molecules, short peptides and proteins	Anti-inflammatory drugs	Exogenous mRNA and its delivery system have the potential to induce inflammation, which significantly affects the stability and translation efficiency of mRNA. Pretreatment with anti-inflammatory drugs can effectively control related adverse reactions and improve the translation efficiency.	Take Dex as an example. The drug molecules have low hydrophobicity and are prone to dissociating from LNPs in the bloodstream, resulting in systemic exposure to corticosteroids.	Systemic exposure to corticosteroid anti-inflammatory drugs poses risks such as triggering diabetes, hypertension, cataracts, and fractures.	Clobetasol and hydrocortisone: Topically apply clobetasol propionate and hydrocortisone ointments at the injection site of the ZIKVac-sa-RNAs vaccine. Hydrocortisone only improves *in vivo* translation on 2- to 3-d after injection, while clobetasol performs better, extending the translation cycle to one week and increasing the overall translation amount of mRNA by 3.5 times.	The anti-inflammatory drugs that have undergone hydrophobic modification are suitable for scenarios where there is a need for systemic exposure to therapeutic proteins, chronic treatment, and self-administration.
	Signaling pathway inhibitors	Theoretically, blocking any step in the signaling pathway of the innate immune response triggered by mRNA can prevent the final degradation of mRNA and the termination of translation.	Signal pathway inhibitors often target only one pathway, and there may be other compensatory pathways that come into play. In many studies, it has been observed that the use of immunosuppressants alone does not produce satisfactory improvement effects.	It is relatively safe to use within the normal concentration range, but using it beyond the normal range will have adverse effects on cells.	ISRIB: Using LNPssPalm as the transfection vector and Luci mRNA as the reporter gene, ISRIB mainly enhances the transfection activity in the early stage (0–6 h), increasing the transfection activity by 1.4 times.	When pathway inhibitors are used in combination, the improvement effects are often additive, making them suitable for scenarios where combined use is required.
	Endosome-related strategies	Promote endosomal escape: Assist the mRNA that has entered the endosome to enter the cytoplasm; Inhibit endosomal recycling: Reduce the number of mRNA NPs transported to the lysosome and increase their concentration in the cytoplasm, thereby improving the translation efficiency of mRNA.	It should have no enhancing effect on the mRNA that enters the cells through non-endocytic pathways (such as membrane fusion technology, etc.).	Take CQ as an example. It usually requires multiple administrations, and its application in areas such as gene editing is limited by systemic toxicity.	PCS: When using Lipofectamine 2000 to transfect HUVEC with EGFP mRNA, the cell transfection rate in the PCS negative group is extremely low, while ∼58% of the cells in the PCS positive group can be transfected.	Strategies related to promoting endosomal escape are of positive significance for the translation of both naked mRNA and mRNA encapsulated in NPs that enter cells through various endocytic pathways.
	RNase inhibitors	Intracellular mRNA also faces degradation by endogenous RNases. To mitigate the impact of RNases during the mRNA translation process, it is necessary to consider using corresponding RNase inhibitors.	Take RNasin as an example. Due to the protein nature of RNasin, it has relatively strict requirements in terms of pH value and operation.	It is relatively safe to use within the normal concentration range. However, if the concentration exceeds the normal range, it will have adverse effects on cells.	RNasin: Supplementing naked sa-RNAs with RNasin before intradermal electroporation can increase the median total luciferase expression by 70-fold and raise the success rate of the drug administration procedure from 33% to 100%.	Take sunitinib as an example. The transient inhibition of innate immunity by sunitinib can enhance the effectiveness of oncolytic virus therapy, thus enabling tumor-bearing animals to recover.

#### 4.1.1 Timing of drug administration

According to research findings and data in the literature, cellular responses to transfection with IVT mRNA involve three stages across different time scales: (1) PKR is activated immediately after transfection, followed by cap-dependent decreases in translation via eIF2α phosphorylation (approximately minutes to hours); (2) activation of NF-κB-induced inflammatory responses inhibits mRNA translation processes (approximately dozens of hours); (3) establishment of type I IFN signal transduction-mediated antiviral immunity and repeated transfections (on a daily basis)[223].Therefore, if the timing of immunosuppressive agent application is mismatched with the immune response elicited by transfection, the desired improvement will likely be affected. For example, in the study by Ohto et al. [223], ISRIB enhanced transfection activity primarily in the early stage of transfection (0–6 h). Conversely, Dex led to increased transfection activity in the middle to late stages of transfection (4–48 h). In the study by Tusup et al. [273], the addition of CQ, which promotes endosomal escape, before or during transfection did not increase IVT mRNA expression. The addition of only low-dose CQ 2 h after transfection resulted in increased mRNA translation. In the study by Zhong et al. [229], the soluble oligonucleotide TLR inhibitors ODN2088 and ODN20958 significantly reduced innate immune responses only when used directly after mRNA delivery; pretreatment did not enhance or prolong this ability.

When an mRNA translation booster is used in combination with an mRNA drug, special attention should be given to the spatiotemporal matching of their pharmacokinetics. For example, Appeldoorn et al. [307] systematically analyzed the pharmacokinetic characteristics of the JAK inhibitor ruxolitinib after oral administration. They reported that as a Class I compound, ruxolitinib has extremely high bioavailability (>95%). After administration of the immediate-release oral formulation, ruxolitinib is rapidly absorbed (with a time to reach the maximum concentration (T_max_) ranging from 0.5 to 6.0 h and an absorption rate constant of 3.43 h^−1^) and exhibits linear pharmacokinetics within the dose range of 5 to 200 mg. When ruxolitinib is used as an mRNA translation booster in combination with an mRNA drug, the rapid absorption (with a median T_max_ of ∼1.5 h) and moderate half-life (roughly 3 to 4 h) of ruxolitinib may lead to a significant time difference between its concentration peak and the translation window of the mRNA drug (which usually reaches peak expression 6 to 24 h after administration). In addition, the high protein binding rate of ruxolitinib may further reduce the free drug concentration, weakening the continuous activation of the translation pathway. It is necessary to enhance the synergistic effect through optimization strategies such as sustained-release formulations and responsive delivery systems to achieve precise drug administration.

#### 4.1.2 Mode of drug administration: standalone use *versus* combined use

Many immunosuppressive agents potently inhibit a specific type I IFN signaling pathway. However, the *in vitro* and *in vivo* outcomes upon their codelivery with mRNA often fail to exhibit significant improvement. This could be attributed to our incomplete understanding of the intricate cellular signaling pathways, as these immunosuppressive agents target only one pathway, while other compensatory pathways may also be involved. Numerous studies have reported that the use of immunosuppressive agents alone does not result in satisfactory improvements. However, when employed in combination, the improvement effect is often cumulative or even multiplied. For example, the TLR inhibitor ODN2088, when used alone, can reduce immediate type I IFN responses, but does not improve translation *in vivo*. However, when combined with topical CQ cream, a cumulative therapeutic effect has been observed [229].

Notably, when formulating strategies for the combined use of mRNA translation enhancers, it is essential to fully consider the inherent characteristics of the mRNA to maximize its therapeutic effect. Consider the different technical platforms introduced previously as examples. Since the viral NSPs carried by the sa-RNA platform may trigger excessive immune activation, priority should be given to translation enhancers that target the activation pathways of viral replicases (such as TLR3/7/8 inhibitors) or immunomodulators that inhibit the signaling of type I IFNs. The dual-molecule delivery system of the ta-RNA platform (replicase mRNA + GOI mRNA) leads to increased immunogenicity, and combinations of multitargeted inhibitors can be considered. Owing to its closed-loop structure, the circRNA platform naturally resists degradation by exonucleases. Therefore, traditional RNase inhibitors (such as RNasin) do not enhance translation.

In addition to the combined application of translation boosters, future research can also focus on achieving a synergistic effect by combining mRNA structural modifications with translation boosters, thus overcoming the limitations of a single strategy and enhancing translation efficiency, duration of action, and tissue specificity. Structural modifications (such as optimization of the 5′ UTR and 3′ UTR, nucleotide chemical modification, and circ-RNA design) can increase the stability of mRNAs and their binding to ribosomes. Translation boosters, on the other hand, can promote the initiation of translation and reduce mRNA degradation. By means of multitarget interventions and rationally designed combination strategies, the key bottlenecks in the expression of mRNAs after delivery can be addressed. The information in this section is supplemented and explained in the Discussion and Conclusion sections of the main text.

#### 4.1.3 Form of drug administration

When designing an mRNA translation booster agent, simple simultaneous administration often yields suboptimal results. A strategic approach based on the distinctive properties of drugs and their sensible introduction is needed to achieve synergy and the precise delivery of the mRNAs and immunosuppressive agents. For example, prophylactic injection of Dex prior to mRNA administration results in certain anti-inflammatory effects. However, it also involves the risks of systemic drug exposure and potential side effects. If Dex is simply mixed into LNPs, it is prone to dissociate during delivery because of its low hydrophobicity [223]. Therefore, researchers have developed a series of ester prodrugs based on the anti-inflammatory structure of Dex. Experimental results have shown that, compared with Dex alone, incorporating Dex into LNPs for mRNA delivery yields better therapeutic effects and significantly reduces the drug dosage and side effects [224].

#### 4.1.4 Intrinsic challenges associated with the mRNA itself

Compared with other nucleic acid drugs, mRNA molecules are larger and more complex, which inherently presents challenges in delivery. Moreover, during their expression, the mRNAs are more susceptible to interference. For example, during delivery, miRNAs may experience mismatch with several nucleotides, even if they do not perfectly complement the target gene. This can hinder the translation and regulatory functions of the protein. Additionally, mRNAs for protein replacement therapy applications require greater molecular integrity to their accurate translation into proteins to exert their effects. Consequently, these mRNAs are somewhat more prone to inefficacy.

### 4.2 Summary

mRNA delivery technology is currently a rapidly developing research area in the field of biopharmaceuticals. Although mRNA therapies have the potential to treat a variety of diseases, their clinical application as non-immunotherapies still face numerous challenges. For example, for chronic conditions that require repeated drug administration (such as genetic diseases, metabolic syndrome, and autoimmune diseases), mRNA therapies encounter difficulties such as decreased long-term expression efficiency, the accumulation of immunogenicity, and a high frequency of administration. Cellular stress (such as endoplasmic reticulum overload) or inflammatory signals can inhibit the activity of translation initiation factors, causing the mRNA expression level to decrease over time. The injection of LNPs may also pose risks of lipid toxicity or organ accumulation (such as in the liver), which limits their long-term use. By using mRNA translation boosters (such as small-molecule enhancers or natural viral proteins), it is possible to synergistically extend the protein expression window, reduce the frequency of administration, decrease the number of administrations, counteract the inhibitory effect of chronic inflammation on translation, and improve the stability of the mRNA after multiple administrations. In conclusion, in the future, it is necessary to balance the benefits and risks of long-term treatment and promote the expansion of mRNA therapies from acute interventions to chronic disease management. mRNA therapies, by virtue of their programmability and rapid response capabilities, also present broad prospects in personalized precision medicine. As a category of molecular tools, translation boosters can be utilized to design personalized immunosuppressive regimens for hypersensitive patients, such as those suffering from autoimmune diseases. By overcoming individual differences, regulating immune responses, and expanding the pertinent patient population, translation boosters could profoundly influence the development trajectory of personalized mRNA therapies. Some progress has been made in mRNA translation booster research, but there is still vast space for exploration. In particular, due to the lack of standardized experimental protocols and measurement criteria in previous studies, there are significant disparities in their results. Therefore, it is particularly important to conduct standardized *in vitro* and *in vivo* experiments. As mentioned above, to quantify the immunomodulatory effects of mRNA translation boosters, *in vitro* or *in vivo* model experiments can be conducted, and various techniques, such as molecular biology experiments and immunological assays, can be employed. For the construction of *in vitro* models, innate immune cells such as primary macrophages or DCs and nonimmune cells such as HEK293T (used for studying the TLR/RIG-I pathway) and pulmonary epithelial cells (serving as the target tissues of mRNA vaccines) can be selected. The mRNA delivery system can also use delivery vectors that are consistent with those used in clinical practice (such as LNPs) to ensure the stability of the mRNA and its intracellular release. To quantify key indicators, the concentrations of proinflammatory factors (IL-6, TNF-α, IFN-α/β and IL-1β) and anti-inflammatory factors (IL-10 and TGF-β) can be detected via ELISA. The phosphorylation levels of IRF3/7 and NF-κB can be detected by methods such as Western blotting. The activity of caspase-1 and the maturation of IL-1β can be detected by Western blotting, flow cytometry, etc. The expression profiles of key pathway genes (such as ISGs and inflammasome components) can be analyzed through RNA-seq.

In addition, certain immunosuppressants or RNA interference technologies that have not yet been applied in the field of mRNA delivery have shown great potential, indicating the need for large-scale drug screening. To ensure that these strategies for improving mRNA expression have broad application prospects and market potential, improving both their cost-effectiveness and efficiency should be considered to achieve the optimal cost‒benefit ratio. The strategies discussed in this article for optimizing mRNA expression efficiency are not only applicable to mRNAs but also serve as a reference for the efficient expression of other nucleic acid drugs, such as siRNAs and shRNAs.

This article reviews the current strategies for optimizing mRNA expression and related translation-enhancing drugs. First, the possible interferences during the mRNA expression process, including degradation in the endosomal recycling pathway and translation inhibition triggered by the mRNA immune response, are discussed. In response to these processes and related signaling pathways, the corresponding translation enhancers are introduced, and the experimental protocols of previous studies are summarized in the form of a table. Finally, the influencing factors and precautions to consider for the application of these mRNA translation-enhancing molecules are discussed, and a comparison of their mechanisms, effectiveness, and usage scenarios is conducted. Future applications of mRNA translation boosters in areas such as chronic diseases and personalized medicine are proposed, with the aim of providing guidance for mRNA research and development.

## Conflicts of interest

The authors declare that there is no conflicts of interest.
